# Role of Efflux in Antibiotic Resistance of *Achromobacter xylosoxidans* and *Achromobacter insuavis* Isolates From Patients With Cystic Fibrosis

**DOI:** 10.3389/fmicb.2022.762307

**Published:** 2022-03-28

**Authors:** Hussein Chalhoub, Stefanie Kampmeier, Barbara C. Kahl, Françoise Van Bambeke

**Affiliations:** ^1^Pharmacologie Cellulaire et Moléculaire, Louvain Drug Research Institute, Université catholique de Louvain, Brussels, Belgium; ^2^Institute of Hygiene, University Hospital Münster, Münster, Germany; ^3^Institute of Medical Microbiology, University Hospital Münster, Münster, Germany

**Keywords:** *Achromobacter*, efflux, target mutation, macrolide, fluoroquinolone, aminoglycoside

## Abstract

*Achromobacter* genus (including *Achromobacter xylosoxidans*, the most prevalent *Achromobacter* species in patients with cystic fibrosis) is poorly susceptible to most conventional antibiotics. Contribution of efflux by AxyABM, AxyXY-OprZ, and AxyEF-OprN and of target mutations were studied in clinical isolates of *A. xylosoxidans* and *Achromobacter insuavis.* Forty-one isolates longitudinally collected from 21 patients with CF were studied by whole-genome sequencing (WGS)-typing, determination of minimum inhibitory concentrations (MICs) of β-lactams, aminoglycosides, colistin, azithromycin, ciprofloxacin, chloramphenicol, and doxycycline, and expression (quantitative RT-PCR) and function (measure of the uptake of a fluorescent substrate) of efflux pumps. WGS-based typing resulted in 10 clusters comprising 2 or 3 isolates and 20 singletons. The efflux activity was high in strains with elevated MICs for amikacin or azithromycin. This work sheds a new light on the impact of efflux and target mutations in resistance of *Achromobacter* to several drugs.

## Introduction

In adults with cystic fibrosis (CF), *Pseudomonas aeruginosa* is one of the main respiratory pathogens, but in recent years, other non-fermenting Gram-negative bacterial species, such as *Stenotrophomonas*, *Burkholderia*, or *Achromobacter* have been increasingly isolated (Cystic Fibrosis Foundation, 2020). This could be due, collectively, to a better eradication of *P. aeruginosa* by aggressive therapies, a lengthening of patients’ life expectancy, and the development of new techniques for bacterial identification. Among these bacteria, *Achromobacter* spp. are ubiquitous environmental microorganisms, also part of the microbiota of the ear and the gastrointestinal tract (Steinberg and Del Rio, 2005). They may become opportunistic pathogens capable of causing a large variety of infections, including endophthalmitis, keratoconjunctivitis, catheter-associated bloodstream infection, endocarditis, pneumonia, meningitis, and peritonitis (Spilker et al., 2012). They are also isolated in patients with CF and cause serious respiratory tract infections (Swenson and Sadikot, 2015; Hoyle et al., 2018). *Achromobacter* can be found in up to 10% of the sputum samples collected from patients with CF, with *A. xylosoxidans* being the most prevalent *Achromobacter* species, identified in 35–80% of the cases (Raidt et al., 2015; Amoureux et al., 2016; Gade et al., 2017; Isler et al., 2020). Its pathogenic role in CF remains unclear, but chronic colonization is associated with a decline in respiratory function (Edwards et al., 2017; Tetart et al., 2019; Marsac et al., 2021) and a higher risk of death or lung transplantation (Somayaji et al., 2017), suggesting the need for an active treatment. At this stage, however, there is no standard treatment protocols for *Achromobacter* infections in CF, and treatment options need to be selected on a case-by-case basis (Isler et al., 2020).

In a clinical perspective, ceftazidime, meropenem, ciprofloxacin, and colistin are representative of the classes for which EUCAST has published MIC distributions against *Achromobacter*.^1^ Extended-spectrum β-lactams also often represent a first option for infections by *Achromobacter* in patients with CF (Swenson and Sadikot, 2015). Inhaled antibiotics (colistin, or tobramycin) proved useful complements to intravenous drugs (Wang et al., 2013). Tetracyclines and chloramphenicol are among the most active agents *in vitro* (Saiman et al., 2001). In addition, temocillin is indicated against *Burkholderia* (Zeiser et al., 2019), and azithromycin is widely used for its anti-virulence and immunomodulatory properties (Cramer et al., 2017), so that patients are also possibly exposed to these drugs even though they have no meaningful activity on *Achromobacter.*

Antibiotic selection remains a real challenge because *Achromobacter* displays not only innate but also frequent acquired multidrug resistance to a wide range of antibiotics commonly used for the management of infections by Gram-negative microorganisms (Traglia et al., 2012; Bador et al., 2013; Isler et al., 2020). Unfortunately, the knowledge of drug resistance mechanisms in this genus is limited. Genes located on mobile genetic elements, which encode β-lactamases or aminoglycoside-modifying enzymes or confer fluoroquinolone resistance, have been reported thus far (Hu et al., 2015; Isler et al., 2020) and contribute to acquired resistance. β-Lactamases can be highly diverse, including extended-spectrum (CTX-M, VEB-1) or AmpC-type (CMY-2, AmpC) β-lactamases hydrolyzing all beta-lactams except carbapenems, and plasmidic (IMP and VIM) carbapenemases (Isler et al., 2020). In addition, five predicted β-lactamase genes have been identified in the chromosome, encoding one class D (*bla*_*OXA*–114_), one class C, two class B, and one class A enzymes (Doi et al., 2008).

Another potential resistance mechanism consists in active efflux through AxyABM, AxyXY-OprZ, or AxyEF-OprN pumps, which seem orthologs of MexAB-OprM, MexXY-OprA, and MexEF-OprN in *P. aeruginosa*, respectively (Bador et al., 2011, 2013; Nielsen et al., 2019; Magallon et al., 2021). The substrate specificity of these pumps and their impact on antibiotic activity is, however, different between these two species.

This study investigated resistance mechanisms in clinical isolates of *A. xylosoxidans* and the closely related species *Achromobacter insuavis*. To this effect, a collection of 41 isolates was assembled longitudinally from patients with CF, which allowed us to consider microevolution in specific genes.

## Materials and Methods

### Isolates, Identification, Whole-Genome Sequencing, and Relatedness

Forty-one successive isolates of *Achromobacter* spp. from sputum samples of 21 patients with CF and cultures remaining positive over prolonged time periods (0.3–11 years interval between the 2 successive samples; mean value, 4.4 years; Table 1) were collected at the CF centers of the University Hospital and Clemenshospital, Münster, Germany (2006–2017). *A. xylosoxidans* ATCC 27061 (Yabuuchi and Oyama, 1971) was used as a reference throughout this work, as being one of the few fully sequenced clinical isolates of *A. xylosoxidans* and for which the three efflux pumps of interest have been functionally characterized among the 6 identified (Hu et al., 2015). In addition, all sequences were compared to those of *A. insuavis* AXX-A, which shows a wild-type phenotype, in particular regarding its susceptibility to ciprofloxacin (Bador et al., 2011). *P. aeruginosa* ATCC 27853 (Fang et al., 2012) was also used as internal control for antimicrobial susceptibility testing. Two *A. insuavis* [Ai: parental clinical isolate, Ai ΔB/ΔY: Ai with deletions in *axyB* (Bador et al., 2011) or *axyY* (Bador et al., 2013)] were provided by Dr. Julien Bador, Department of Bacteriology, University Hospital of Dijon, Dijon, France. AX-08 and its deletion mutant in *axyE* were provided by Niels Norskov-Lauritsen, Aarhus University, Aahrus, Denmark (Nielsen et al., 2019).

**TABLE 1:** Individual MICs for the reference strains and the 41 isolates.

Patient’s number	Isolates^a^			MIC (mg/L)^b^																	Gene expression level		
		Collection Date	Sampling interval (years)	CAZ	CAZ + AVI (32 mg/L)	MEM	PIP + TZB (4mg/L)	TIC	TIC + TZB (32 mg/L)	TIC + AVI (32 mg/L)	CST	AMK	AMK + BER (128 mg/L)	TOB	CIP	TMO	AZI	AZI + BER (128 mg/L)	CHL	DOX	*axyB*	*axyY*	*axyF*
–	ATCC 27061	2018		8	4	4	0.5	2	2	2	4	1,024	64	256	4–8	512	128	16	64	16	1	1	1

–	AXX-A			8	4	0.25	2	0.5	0.5	0.25	2–4	128	16	128	1–2	1,024–2,048	64	64	16	4	1.96	1.22	ND
–	AXX-A-Δ-*axyB*			4	2	0.125	16	512	0.25	0.125	2–4	128	16	128	1	256–512	64	64	4	2	3.08	1.80	ND
–	AXX-A-Δ-*axyY*			8	4	0.125	16	512	2	0.5	2–4	32	4	32–64	1	1,024–2,048	16	128	16	2	2.13	6.96	ND

–	AX08			2	2	0.25	1	2	2	2	1–2	128	16	128	2	256	32–64	8	16	16	4.57	3.96	3.40
–	AX08-Δ*axyE*			2	2	0.25	1	256	4	2	1–2	128	16	128	1	256	32–64	8	16	8	5.73	3.81	4.98

1	1.1	Dec 19, 2011	3.7	4	2	0.5	0.5	2	2	2	8	512	64	64	8	256	256	32	32	16	2.28	2.72	8.11
	1.7	Sep 2, 2015		32	32	16	> 2,048	>2,048	> 2,048	>2,048	4	> 2,048	128	1,024	32	> 2,048	256	16	32	16	3.16	4.15	0.57
2	2.1	Nov 30, 2016	0.8	4	nd	0.5	0.5	2	2	2	8	512	32	128	4	256	128	16	32	16	0.49	0.17	0.80
	2.3	Sep 20, 2017		4	nd	0.125	0.5	2	2	2	16	512	32	128	4	256	128	16	16	8	0.60	0.16	0.75
3	3.1*^d^*	Jan 5, 2012	–	4	nd	0.125	2	128	128	128	1	128	16	128	1	> 2,048	128	16	8	1	0.82	0.04	0.68
4	4.1	Feb 11, 2010	7.5	64	16	4	0.5	4	4	4	1,024	> 2,048	256	512	4	2,048	> 2,048	>2,048	8	16	2.43	1.72	2.23
	4.15	Aug 21, 2017		64	32	4	32	32	32	8	> 2,048	>2,048	256	512	16	2,048	> 2,048	>2,048	8	16	1.67	1.34	2.20
5	5.1	Aug 17, 2007	8.5	4	2	4	2	8	8	8	16	2,048	32	256	16	> 2,048	512	128	32	8	1.35	0.64	0.70
	5.12	Jan 12, 2016		256	128	256	> 2,048	>2,048	> 2,048	>2,048	8	2,048	64	128	32	> 2,048	>2,048	> 2,048	32	16	2.25	0.48	0.70
6	6.1^c^	Aug 17, 2006	11	256	32	512	> 2,048	>2.048	> 2.048	64	1,024	> 2,048	64	256	16	> 2,048	128	8	8	16	4.08	1.98	2.44
	6.14^c^	Sep 25, 2017		256	32	256	> 2,048	>2,048	> 2,048	64	1	16	16	4	16	> 2,048	8	8	16	4	4.19	0.11	1.73
																							
7	7.1	Aug 28, 2014	2.2	4	nd	0.25	1	2	2	2	**4**	**512**	**64**	**256**	**4**	512	128	16	**64**	**16**	0.96	0.47	1.47
	7.3	Nov 23, 2016		4	nd	4	0.25	2	2	2	**256**	**32**	8	**16**	**4**	256	128	16	**64**	**16**	1.65	0.65	2.07
8	8.1	Apr 21, 2010	4.7–5.5	**16**	8	2	8	4	4	4	**2,048**	** > 2,048**	**1,024**	** > 2,048**	**8**	>2,048	> 2,048	256	**16**	**8**	2.66	4.63	0.65
	8.6	Dec 3, 2014		**256**	**256**	**16**	**32**	**256**	**256**	**256**	**2,048**	** > 2,048**	**1,024**	** > 2,048**	**32**	>2,048	> 2,048	256	**16**	**8**	2.13	3.64	0.71
	8.7	Sep 3, 2015		**256**	**256**	**64**	**512**	**256**	**256**	**256**	**2,048**	** > 2,048**	**>2,048**	** > 2,048**	**32**	>2,048	> 2,048	256	**32**	4	2.03	3.53	0.71
9	9.1^c^	Jul 13, 2010	2–3.3	4	nd	1	1	4	4	4	**128**	**32**	8	**8**	**4**	512	64	8	**64**	**16**	1.03	0.26	6.53
	9.6^c^	Jun 19, 2012		4	nd	**64**	1	**1,024**	**1,024**	8	**256**	**128**	16	**16**	**8**	1,024	512	16	**512**	**32**	0.82	0.28	8.11
	9.8^c^	Nov 19, 2013		4	nd	4	0.5	2	2	1	**256**	**256**	**64**	**16**	**8**	256	> 2,048	32	**32**	**16**	5.17	4.87	5.82
10	10.1^c,d^	Feb 18, 2010	1.5	8	nd	**16**	**> 2,048**	**1,024**	**1,024**	**1,024**	**256**	16	8	4	**4**	> 2,048	32	32	**16**	**8**	1.31	0.08	0.73
	10.3^c,d^	Jul 26, 2011		8	nd	**128**	**> 2,048**	**1,024**	**1,024**	**128**	**4**	**64**	**32**	**32**	**128**	> 2,048	256	32	**512**	**32**	1.11	0.44	63.05
11	11.1	Dec 2, 2010	4.9	**256**	**256**	**128**	** > 2,048**	**2,048**	**2,048**	**2,048**	** > 2,048**	**>2,048**	** > 2,048**	**512**	**16**	> 2,048	>2,048	> 2,048	**32**	**8**	1.22	0.64	0.60
	11.10	Nov 10, 2015		**256**	**256**	**512**	** > 2,048**	**2,048**	**2,048**	**2,048**	** > 2,048**	**>2,048**	** > 2,048**	**1,024**	**16**	> 2,048	>2,048	> 2,048	**16**	**8**	1.86	3.02	0.43
12	12.1	Feb 1, 2008	8.5	4	nd	0.5	0.5	**512**	**512**	**512**	**16**	**256**	**32**	**256**	**4**	> 2,048	64	16	**32**	**16**	0.80	0.19	0.77
	12.9	Sep 12, 2016		4	nd	**32**	** > 2,048**	**512**	**512**	**512**	**32**	**256**	**32**	**128**	**4**	> 2,048	64	16	**32**	**16**	0.74	0.21	0.53
13	13.1	Aug 25, 2009	8.1	8	4	4	** > 2,048**	4	2	1	**16**	**512**	**32**	**256**	**16**	> 2,048	128	16	**32**	**16**	0.87	0.98	2.42
	13.15	Sep 20, 2017		**128**	**128**	**32**	** > 2,048**	**128**	**128**	**128**	** > 2,048**	512	32	**256**	**8**	> 2,048	128	8	**32**	**16**	1.69	0.75	1.75
14	14.2	Sep 30, 2014	1.8	**1,024**	**128**	**8**	** > 2,048**	**64**	**64**	**64**	**4**	256	64	**128**	**8**	> 2,048	>2,048	> 2,048	**16**	4	0.76	1.58	0.31
																							
																							
	14.4	Jul 6, 2016		2,048	2,048	32	> 2,048	>2,048	> 2,048	>2,048	2	1,024	64	128	16	> 2,048	>2,048	> 2,048	16	4	1.91	2.84	0.40
15	15.1^c,d^	Jul 1, 2009	4.1	4	nd	1	> 2,048	512	512	256	4	64	16	16	4	> 2,048	16	16	4	≤ 0.5	0.32	0.10	0.55
	15.4^c,d^	Aug 7, 2013		2,048	2,048	4	> 2,048	>2,048	> 2,048	>2,048	4	256	32	128	4	> 2,048	16	16	4	0.5	0.37	0.14	0.60
16	16.1	Nov 8, 2016	1.9	4	nd	1	0.5	4	4	2	4	16	4	8	16	512	16	16	512	1	0.96	0.23	201.60
	16.6	Sep 20, 2017		4	nd	0.5	0.5	4	4	2	2	16	4	8	16	512	16	16	512	1	0.96	0.24	119.89
17	17.1^c^	Dec 18, 2014	–	1,024	64	128	64	> 2,048	>2,048	64	64	1,024	32	> 2,048	16	>2,048	> 2,048	>2,048	32	16	1.09	1.11	0.90
18	18.1^c^	Aug 25, 2009	1.5	8	nd	2	1	2,048	2,048	2,048	2	128	16	64	4	2,048	64	16	32	16	0.65	0.11	0.98
	18.3^c^	Feb 15, 2011		8	nd	64	2	2,048	2,048	2,048	4	128	16	64	8	2,048	64	16	32	16	1.45	0.92	1.17
19	19.2^c^	Jan 5, 2010	0.35	4	nd	4	1	1,024	1,024	4	0.5	256	32	32	32	1,024	128	16	512	16	1.39	0.40	16.20
	19.5^c^	May 10, 2010		2	nd	4	0.5	1,024	1,024	4	1	8	8	4	32	1,024	64	8	512	16	1.27	0.14	8.89
26	26.3	Aug 16, 2017	–	4	nd	2	2	4	4	4	2	256	32	64	4	1,024	128	16	64	8	0.72	0.30	0.57
27	27.1^d^	Feb 18, 2010	3.75	2	nd	0.25	1	> 2,048	>2,048	> 2,048	2	64	16	64	2	512	64	16	16	0.5	1.21	0.11	1.06
	27.3^d^	Nov 12, 2013		2	nd	0.25	1	2	2	4	4	256	32	64	4	512	64	16	32	2	1.27	0.12	1.13
																							

*^a^Strain numbering: first figure corresponds to the patient; second figure correspond to the isolate number in each patient (late isolate collected over the period of sampling).*

*^b^Values in bold are above the CLSI breakpoint (when available; see Table 1 for values).*

*^c^Gray background highlights MICs of strains in which cephalosporinase activity was phenotypically detected.*

*^d^A. insuavis.*

Strain relatedness was analyzed and represented by a minimum spanning tree, after whole genome sequencing (WGS). Isolates were compared *via* WGS-based typing using the Illumina MiSeq platform (Illumina Inc., San Diego, CA) (Dekker and Frank, 2016). After quality trimming, coding core genome regions were compared in a gene-by-gene approach (core genome multilocus sequence typing, cgMLST) using the SeqSphere + software version 6.0.2 (Ridom GmbH, Münster, Germany) and *A. xylosoxidans* ATCC 27061 within an *ad hoc* scheme as a reference sequence (GenBank accession number LN831029.1). SeqSphere + software was used to display the clonal relationship in a minimum-spanning tree. Threshold defining close genetic relation was set after plotting allelic changes over time of strains derived from each and the same patient. Species identification was performed using the *nrdA* gene data (Spilker et al., 2012). These were first extracted with the help of SeqSphere + from the WGS data *in silico* and thereafter subjected to *nrdA*_765 typing available by PubMLST. On the basis of all *nrdA* genes, a phylogenetic analysis was performed using software MEGA 11 (Tamura et al., 2021) in which the evolutionary history was inferred from a Maximum Likelihood method and General Time Reversible model (Nei and Kumar, 2000) implying 1,000 bootstrap replications. The sequences of *rrl* encoding 23S rRNA, of *rpl4* and *rpl22* ribosomal genes and of *gyrA*, *gyrB*, *parE*, and *parC* were compared to the reference strains AXX-A and ATCC 27061 by pairwise sequence alignment.

### Antimicrobial Susceptibility Testing

MICs were determined by broth microdilution according to CLSI guidelines (Clinical and Laboratory Standards Institute, 2020) for the following antibiotics (potency and origin): amikacin (Amukin 500 mg/2 ml for injection, S.A. Bristol-Myers Squibb, Belgium), azithromycin (100%, SMB, Brussels, Belgium), ceftazidime (2 g for IV injection, 72.5%, PAN Pharma, Luitré, France), chloramphenicol (98%, Sigma-Aldrich), ciprofloxacin (98%, Fluka, Sigma-Aldrich), colistin sulfate salt (79.6%, Sigma-Aldrich), doxycycline hyclate (86.6%, Sigma-Aldrich), meropenem (500 mg powder for solution for injection or infusion, 92%, Hospira UK Ltd., Hurley, United Kingdom), piperacillin (94.2%, Sigma-Aldrich, Maryland Heights, MO), temocillin (84%, Eumedica Pharmaceuticals, Manage, Belgium), ticarcillin disodium salt (85.2%, Sigma-Aldrich), and tobramycin (100%, Teva, Wilrijk, Belgium). Tazobactam sodium salt (92.4%, Cubist Pharmaceuticals, Lexington, MA) and avibactam (99.6%, AstraZeneca Pharmaceuticals, Waltham, MA) were used as inhibitors for β-lactamases. Berberine (chloride hydrate, 82.1%; Sigma-Aldrich), known to attenuate the MexXY-OprM/OprA-mediated aminoglycoside resistance in *P. aeruginosa* (Morita et al., 2016), was used to reduce AxyXY-OprZ activity.

### Uptake of *N*-Phenyl-1-Naphthylamine

The uptake of the lipophilic probe *N*-phenyl-1-naphthylamine (NPN) was measured following the general methodology described previously (Lomovskaya et al., 2001). In brief, 10 ml of bacterial culture in exponential growth phase (OD_620n*m*_ 0.6) was harvested by centrifugation (3,000 *g*; 10 min) and resuspended in buffer (NaCl, 110 mM; KCl, 7 mM; NH_4_Cl, 40 mM; NA_2_HPO_4_, 0.4 mM; Tris base, 52 mM; glucose, 0.2%; pH 7.5 adjusted with HCl). NPN was added at a final concentration of 10 μM, and cells were incubated 10 min at 37°C; then, 200-μl aliquots were dispensed in standard 96-well plates. The fluorescence signal (excitation/emission wavelengths, 340/410 nm) was measured on a Spectramax® multiplate reader (Molecular Devices, Sunnyvale, CA). The protonophore carbonyl cyanide m-chlorophenyl hydrazine (CCCP, 97%, Sigma-Aldrich; final concentration, 100 μM) was used as a positive control; buffer solutions containing 10 μM NPN without cells or cells without NPN were used as blanks.

### Phenotypic Screening of β-Lactamases

Cephalosporinase and carbapenemase activity was detected using the ESBL NDP/carba NP test (Nordmann et al., 2012a,b), adapted by using cefotaxime or imipenem (3 mg/ml) as substrates. *A. xylosoxidans* ATCC 27061 and *P. aeruginosa* ATCC 27853 were included as negative controls and clinical isolates of *P. aeruginosa* expressing IMP-13 or VIM-2 metallo-β-lactamases or *Klebsiella pneumoniae* expressing OXA-48, as positive controls.

### Quantification of Efflux Gene Expression

The expression levels of a*xyB*, *axyY*, and *axyF* (encoding the inner membrane protein of AxyABM, AxyXY-OprZ, or AxyEF-OprN pumps, respectively) were quantified by real-time PCR, relative to those measured for the reference strain ATCC 27061. RNA was extracted (Invitrap Spin Cell RNA Mini Kit, 1061100300, STRATEC, Birkenfeld, Germany) from log-phase cultures (OD_620 *nm*_, around 0.7) and treated by DNase (TURBO DNA-free™ Kit, AM1907, Thermo Fisher Scientific, MA). The absence of DNA contamination was checked by performing a PCR on purified RNA, which did not amplify any residual material. cDNA was synthesized (Transcriptor First Strand cDNA Synthesis Kit, 04379012001, Roche, LifeSciences, Penzberg, Germany). A real-time PCR was performed on a CFX-96 machine (BIORAD, Hercules, CA) using SsoAdvanced™ Universal SYBR® Green Supermix, #1725271, the primers described in Supplementary Table 1, and 16S rRNA as housekeeping gene. Triplicates measurements were repeated in two independent experiments.

### Statistical Analyses

Descriptive statistics and graphs were produced using GraphPad Prism version 8.4.3 (GraphPad Software, San Diego, CA) or JMP Pro v.14 (partition tree analysis; SAS Institute Inc., Cary, NC).

## Results

### Genetic Relation of Strains

The clonal relationship of the 41 isolates (from 21 patients, including 18 pairs/triplets of early/late isolates) was analyzed by WGS typing. Species identification was confirmed *via* extracted *nrdA* gene data, resulting in *A. xylosoxidans* (isolates from patients 1, 2, 4, 5, 6, 7, 8, 9, 11, 12, 13, 14, 17, 18, 19, and 26) or *A. insuavis* (isolates from patients 3, 10, 15, 16, and 27) according to the *nrdA*_765 typing scheme (Supplementary Figure 1A). As the isolates of patient 18 (18.1 and 18.3) are distantly related to all known species according to the phylogenetic analysis, these isolates may represent a new species within the genus *Achromobacter.*

After plotting allele changes in patient isolate pairs per year (range from 0.5 in pairs from patient 12 to 215.5 in pair from 11), the cutoff value for a very close genetic relation resulted in ≤ 20 alleles and for a close genetic relation in ≤ 57 alleles change per year. Results of WGS typing of the 41 *Achromobacter* isolates were displayed in two minimum spanning trees (MST) separated by species. Considering the allelic differences over time, MST of *A. xylosoxidans* strains resulted in 12 clusters (Supplementary Figure 1B-A, black borders) comprising 2, 3, or 5 very closely related or closely related isolates and 3 singletons *via* cgMLST algorithm of 5,778 target genes. Ten clusters harbored strains isolated from one patient and two clusters strains derived from two patients, illuminating the possibility of a patient-to-patient cross-transmission (patients 9 and 19; patients 7 and 26) of *A. xylosoxidans*. MST of *A. insuavis* strains resulted in four clusters (Supplementary Figure 1B-B, black borders) comprising two or three very closely related isolates. Three of these clusters harbored strains isolated from one patient, while one cluster included strains from two patients (patients 3 and 27), also giving hint for a patient-to-patient cross-transmission. Both MST reference genomes (GenBank accession numbers LN831029.1 and GCA_003096315.1) were not genetically related to any genotype from patient isolates of this study.

### Minimum Inhibitory Concentrations, Resistance Profile

MICs were measured for antibiotics commonly used in CF and belonging to classes described as substrates for efflux in other Gram-negative bacteria (Table 1 for individual values and Table 2 for a summary).

**TABLE 2:** Antimicrobial susceptibility to different antibiotics among the 41 clinical isolates [including 18 pairs of successive isolates with 1–11 year’s interval between the early (E) and late (L) sample] of *Achromobacter* spp.

Antibiotics^a^	CLSI susceptibility	Whole collection			Median of MICs (mg/L) from pairs		Geom. Mean of MICs (mg/L) from	
	breakpoints (mg/L)^d^ (S ≤)	(*n* = 41)			of isolates (*n* = 36) (25–75% percentile)		pairs of isolates (*n* = 36) (with 95% CI)	
		% S	MIC_50_	MIC range	E	L	E	L
CAZ*	8	61	4	2–2,048	4 (4–40)	20 (4–256)	12 (5–29)	32 (10–104)
MEM*	4	59	4	0.125–512	2 (0.5–6)	24 (4–96)	3 (1–7)	14 (4–47)
**TZP**	16	54	2	0.25– >2,048	1 (0.5–4,096)	2,304 (0.5–4,096)	15 (2–119)	91 (11–764)
**TIC**	16	37	256	2– >2,048	36 (4–1,536)	768 (3–4,096)	59 (13–274)	196 (41–927)
**TIC + TZB^b^**	16	37	256	2– >2,048	36 (4–1,536)	768 (3–4,096)	59 (13–274)	196 (41–927)
**TIC + AVI^b^**	16	46	64	1– >2,048	6 (2–768)	128 (3–2,048)	29 (7–118)	78 (18–343)
**CST**	2	22	8	0.5– >2,048	12 (4–640)	6 (3–1,152)	26 (7–102)	29 (6–131)
**AMK**	16	12	256	8– >2,048	384 (64–3,072)	256 (48–3,072)	335 (132–851)	299 (108–829)
**AMK + BER** ^c^	16	32^e^	32	4– >2,048	32 (16–64)	32 (16–96)	45 (19–106)	53 (21–134)
**TOB**	4	7	64	4– >2,048	128 (24–256)	128 (16–384)	98 (41–233)	87 (33–228)
CIP*	1	2	8	1–128	6 (4–16)	16 (4–32)	7 (5–10)	13 (8–21)
**TMO**	NA^f^	NA	2,048	256– >2,048	3,072 (512–4,096)	4096 (512–4,096)	1625 (946–2,795)	1,756 (1,001–3,078)
**AZI**	NA	NA	128	8– >2,048	128 (64–2,304)	128 (64–4096)	196 (77–497)	256 (86–766)
**AZI + BER** ^c^	NA	NA	16	8– >2,048	16 (16–192)	16 (16–2176)	53 (18–156)	62 (18–206)
**CHL**	8	15	32	4–512	32 (16–32)	32 (16–64)	31 (17–55)	38 (20–73)
**DOX**	4	27	16	≤0.5–32	16 (8–16)	16 (4–16)	8 (4–14)	8 (5–14)

*^a^CAZ, ceftazidime; MEM, meropenem; TZP, piperacillin/tazobactam; TIC, ticarcillin; CST, colistin; AMK, amikacin; TOB, tobramycin; CIP, ciprofloxacin; TMO, temocillin; AZI, azithromycin; DOX, doxycycline; CHL, chloramphenicol; TZB, tazobactam; AVI, avibactam; BER, berberine.*

*^b^Used at 32 mg/L.*

*^c^Used at 128 mg/L [1/4 MIC; pH of the medium remaining stable (7.3)].*

*^d^Breakpoints for “other non-Enterobacterales.”*

*^e^Percentage calculated if considering AMK breakpoints.*

*^f^NA: not available (no breakpoints set for temocillin and azithromycin).*

*Significant differences in MIC distribution between E and L isolates (Wilcoxon matched-pairs signed-rank test) for CAZ (p: 0.03), MEM (p: 0.01), and CIP (p: 0.01).

**FIGURE 1:**
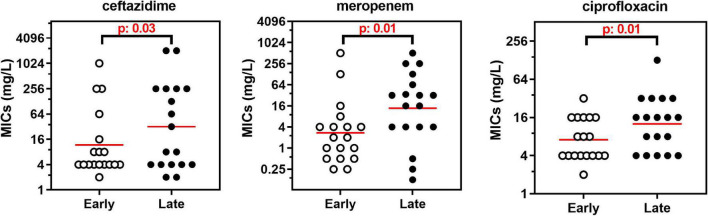
MICs of 3 antibiotics in the 18 pairs of isolates collected successively in the same patients with an interval time ranging from 1 to 11 years. The graphs show the individual MIC for each isolate, with the geometric mean rep-resented by the red horizontal line. Only antibiotics for which a significant difference between the successive isolates (Early vs. late) was observed are illustrated (see Table 1). Statistical analysis: Wilcoxon matched-pairs signed-rank test.

Susceptibility was higher for β-lactams (37–61%) than for doxycycline (27%), colistin (22%), chloramphenicol (15%), aminoglycosides (7–12%), and ciprofloxacin (2%). High MIC_50_ were observed for temocillin and azithromycin (no susceptibility breakpoint set). Broad ranges of MIC values were observed for all drugs, and MIC_50_ were higher than the concentrations reachable in the serum of treated patients, except for ceftazidime, meropenem, and piperacillin/tazobactam. No systematic difference in MICs could be evidenced between strains identified as *A. xylosoxidans* or *A. insuavis*, but the number of strains in each species was too small to draw meaningful conclusions in this respect. When comparing longitudinally the MIC distributions in the 18 pairs/triplets of isolates, a significant increase was observed for ceftazidime, meropenem, and ciprofloxacin between early and late isolates (Wilcoxon matched-pairs signed-rank test: p: 0.03, 0.01, p: 0.01, respectively; see the changes in the median and geometric mean values for MICs of all drugs in early and late isolates in Table 2 and the detailed analysis for drugs showing a significant loss in susceptibility between early and late isolates in Figure 1).

### Phenotypic Screening of β-Lactamase Activity

The percentage of susceptibility to ticarcillin (37%; 15/41) was not modified in the presence of tazobactam, but increased to 46% (19/41) in the presence of avibactam (Table 2). Among the 16 isolates resistant to ceftazidime (MICs, 16–2,048 mg/L), MICs were decreased by 2.2 twofold dilutions on average by avibactam, with only one isolate (8.1; MIC, 16 mg/L) regaining susceptibility to ceftazidime (MIC with avibactam, 8 mg/L; Table 1). All isolates were also screened for β-lactamase activity using the NDP/carba NP phenotypic test. Fourteen isolates were displaying cephalosporinase activity (degradation of cefotaxime), among which 12 were resistant to ticarcillin, 7 to piperacillin-tazobactam, to ceftazidime, and 7 to meropenem, respectively (Table 1 for an identification of these isolates). No carbapenemase activity was detected in the whole collection.

### Efflux Pumps and Influence on Antibiotic Activity

We first showed a minor impact of deletion of each efflux pumps in reference strains on the MIC of the whole panel of antibiotics (Table 1). We therefore rather examined whether the expression levels of *axyB*, *axyY*, and *axyF* encoding the inner membrane protein of each of the three main RND efflux pumps were variable among isolates. The expression levels of these genes in early and late isolates are compared in Supplementary Figure 2. Supplementary Figure 3 shows that there is a significant correlation between the level of expression of *axyB* and the MICs of amikacin, azithromycin, and meropenem, between the expression level of *axyY* and the MICs od amikacin (± berberine), tobramycin, azithromycin, and colistin, and between the expression level of *axyF* and the MICs of chloramphenicol (see Supplementary Table 2 for statistical analyses).

**FIGURE 2:**
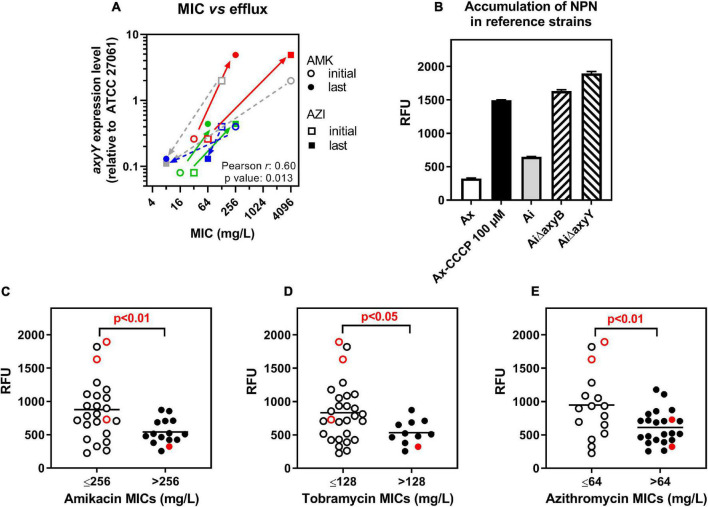
Influence of active efflux on amikacin, tobramycin and azithromycin activity. **(A)** Correlation between the MIC of amikacin (AMK) or azithromycin (AZI) and the level of expression of *axyY* (relative to that in ATCC 27061) in 4 pairs of isolates, represented by different colors. Plain arrows joint data points of pairs for which the late isolate was more resistant [from patient 9 (red) and 10 (green)]; dotted arrows, those for which the late isolate was more susceptible [from patient 6 (gray) and 19 (dark blue)]. Pearson coefficient and *p*-values of the correlation are given. **(B)** Accumulation of the fluorophore NPN in reference strains (Ax: *A. xylosoxidans* ATCC 27061, Ai: *A. insuavis*), mutants (AiΔB: *A. insuavis* lacking *axyB* gene, AiΔY: *A. insuavis* lacking *axyY* gene), or in the presence of CCCP (100 μM) used as a positive control in Ax to reduce the activity of efflux systems, after 10 min of incubation at 37°C. Data are means ± SD of three independent determinations and are expressed in arbitrary fluorescence units (standardized initial inoculum for all strains). **(C–E)** Accumulation of NPN in the same conditions in reference and clinical isolates stratified as a function of their MICs for amikacin, tobramycin, or azithromycin. Red open-closed symbols: reference strains; black open-closed symbols: clinical isolates. Statistical analysis: partition tree to determine the MIC value splitting the distribution in 2 with the highest Logworth (-log *p*-value) value (*p*-value indicated on the graphs). The horizontal line corresponds to the mean value.

The MIC of amikacin and azithromycin was then measured in the whole collection in the presence of the MexXY efflux attenuator berberine (Morita et al., 2016). At 1/4 MIC, berberine decreased these MICs, causing a reduction of the MIC_50_ of 2 and 3 doubling dilutions, respectively (Tables 1, 2). Yet, no changes in the MIC were observed for a few strains with low or high MICs. Thus, amikacin activity remained unaffected by berberine in 3/9 isolates with MIC > 2,048 mg/L (isolates 8.7, 11.1, 11.10) and 1/1 and 1/4 isolates with MIC of 8 and 16 mg/L (isolates 19.5 and 6.14, respectively), respectively (Supplementary Figure 4A). Azithromycin activity was not improved by berberine in 8/12 isolates with MIC > 2,048 mg/L and 6/6 isolates with MIC ≤ 32 mg/L (Supplementary Figure 4B). In four pairs of isolates, the MICs of amikacin and azithromycin changed in parallel between the early and late isolates (late isolate more resistant for patients 9 and 10 or more susceptible for patients 6 and 19; see Table 1), giving us the opportunity to examine the relationship between this change in MIC and the expression levels of *axyB* or *axyY*. No systematic correlation was observed between MICs and the expression level of *axyB* (not shown) but well between MIC values and the expression levels of *axyY* (Figure 2A). We also evaluated the efflux activity in individual strains by measuring the fluorescence signal associated with the incorporation in bacterial membranes of *N*-phenyl-1-naphthylamine (NPN), a well-established substrate for efflux (Ocaktan et al., 1997). To validate this approach, we first showed that NPN accumulation was markedly increased in mutants of an *A. insuavis* reference strain deleted in *axyB* or *axyY* and in an *A. xylosoxidans* reference strain incubated with CCCP as compared to their wild-type counterparts (Figure 2B). NPN fluorescence was then measured in clinical isolates, with data stratified according to antibiotic MICs (Figures 2C–E). Isolates with low MICs for aminoglycosides or azithromycin showed significantly higher NPN accumulation (MIC threshold set at 256, 128, and 64 mg/L for amikacin, tobramycin, and azithromycin, respectively, by partition analysis).

### Ribosomal Mutations and Decreased Azithromycin Activity

Fourteen isolates in this collection showed azithromycin MICs > 256 mg/L (2 isolates with a MIC of 512 mg/L and 12 isolates with a MIC > 2,048 mg/L, respectively; see Table 1). Ribosomal mutations in *rpl4*, *rpl22*, and *rrl* genes were therefore searched in these isolates and in 17 representative isolates with lower MICs (8–256 mg/L). The sequences of the isolates were first compared to those of *A. xylosoxidans* ATCC 27061. However, this reference strain does not show a wild-type phenotype (elevated MICs to some antibiotics; see Table 1). Since there is no fully sequenced wild-type *A. xylosoxidans*, we rather decided to compare all sequences to that of *A. insuavis* AXX-A which is more susceptible to meropenem, ticarcillin, aminoglycosides, ciprofloxacin, chloramphenicol, and doxycycline; Table 1). All the data are compiled in Supplementary Table 3 together with azithromycin MICs (± berberine) and *axyB* or *axyX* gene expression levels. In this Supplementary Table 3, we specifically identify in blue color the mutations that distinguish *A. xysoloxidans* ATCC 27061 from *A. insuavis* AXX-A, and isolates are ordered to show close from one another those which share the same mutations, classified according to their MIC. Three silent mutations (T96C, T123C, and T411C) were commonly observed in *rpl4*, including in *A. xysoloxidans* ATCC 27061, while a few others were only seen in clinical isolates (C219G, C330T, C444T, and C414T). Two mutations leading to the replacement of an uncharged amino acid by a charged amino acid (Q65R and G69R) were detected in isolates with an MIC of 512 mg/L (isolates 5.1 and 9.6). In *rpl22*, silent point mutations (T78C, T189C) were seen in *A. xysoloxidans* ATCC 27061 and in several clinical isolates. A missense conservative *rpl22* T134C (V45A) mutation was observed in the reference ATCC 27061 and in many isolates. It is most likely not related to resistance, as it was also found in isolates with an MIC of azithromycin of ≤ 64 mg/L (*viz.*, isolates 27.1, 27.3, or 10.1 and ATCC 27061). A series of mutations were commonly observed in *rrl*, independent of their azithromycin MIC in the presence of berberine (16 to > 2,048 mg/L), most of them being identified when comparing *A. xysoloxidans* ATCC 27061 and *A. insuavis* AXX-A. Among the 12 isolates with azithromycin MICs > 2,048 mg/L, 11 showed specific mutations in the *rrl* gene (A1284G, T1325C, A2043T, A2043G, A2044G, or C2596T). No specific mutation was identified in isolate 17.1. *axyY* expression was globally low in isolates that were more susceptible to azithromycin than the reference strain or in isolates presenting mutations in *rpl4* or *rpl22*. Conversely, *axyY* expression was variable among isolates harboring mutations in *rrl*, but at least one of the two efflux-associated genes was overexpressed in all isolates with MICs > 2,048 mg/L, except for isolates 17.1 and 11.1.

### Mutations in Fluoroquinolone Targets and Resistance to Ciprofloxacin

Ciprofloxacin displayed marginal activity against this collection, with MIC ranging from 1 to 32 mg/L. As *A. xylosoxidans* shows an elevated MIC to ciprofloxacin (4 mg/L), its sequence was first compared with that of *A. insuavis* AXX-A. Several missense mutations (shown in green) in the sequence of *gyrA*, *gyrB*, *parC*, and *parE* were detected and an insertion of 3 amino acids at the end of the *gyrA* sequence, which were also found in other isolates with an MIC of 4 mg/L (Supplementary Table 4). Of note, the isolate 10.1, with an MIC of 4 mg/L and a basal expression of *axyF*, shows the same sequence for the four genes as *A. insuavis* AXX-A, which may suggest that the mutations seen in *A. xylosoxidans* ATCC 27061 are not necessarily explaining its elevated MIC. The isolate 3.1 (susceptible; MIC = 1 mg/L) showed only a few differences with the sequence of *A. insuavis* AXX-A, some of them being also found in *gyrB* or *parC* of *A. xylosoxidans* ATCC 27061. Other mutations were specifically identified in clinical isolates with ciprofloxacin MIC ≥ 4 mg/L, namely, T527S and M706V in *gyrA* (isolates from patients 18 and 8), N11T, G12S, N592S or N592G, S609A, A631T, A633S, and A634T in *gyrB* (isolates from patients 18, 15, and 5); V417L, E441Q, S482T, and K764R in *parC* (isolates from patients 9, 19, and 18); and V41I, A150T, and T593S in *parE* (isolate from patient 18). Lastly, other missense mutations, located exclusively in *gyrA* (Q83L, D87N, L454M, T881M; patients 4, 8, 16, 17) and *gyrB* (I683V; patient 5), were detected only in isolates with MICs ≥ 16 mg/L. Among all these mutations, only Q83L and D87N were located in the QRDR for *gyrA*. *axyF* expression was low in most of the isolates, except 10.3, 16.1, and 19.2 and associated with specific target mutations in 16.1 and 19.2. Of note, isolates 9.1 and 19.2 showed the same mutations in *parC* but different MICs depending on the level of expression of *axyF*. Noteworthy, the only isolate in the collection with a MIC of 128 mg/L (10.3, patient 10) did not show any specific QRDR mutation but rather a particularly high level of expression of *axyF* as compared to the previous isolate from the same patient (10.1). No other mutations were found in the isolates that are not shown in Supplementary Table 4.

## Discussion

This study highlights a major, and sometimes unexpected, role of efflux and target mutations in the resistance of *Achromobacter* isolated from patients with CF to specific antibiotic classes, thanks to the exploitation of a collection containing in majority longitudinal pairs of isolates from the same patients.

Whereas WGS-based typing has been successfully set up, a public cgMLST scheme for interpretation of genetic relatedness is lacking. Hence, an *ad hoc* scheme was established here [≤ 20 allele difference (de Been et al., 2015)]. Based on this strict criterion, only about half of the isolates kept a very high degree of relatedness over time, possibly indicating that genetic adaptations have taken place under selective pressure during antibiotic treatment. Although not powered as an epidemiological survey, our study shows that, among the tested drugs, the most active ones belong to the class of β-lactams, in accordance with previous reports from France (Amoureux et al., 2013; Dupont et al., 2017), Italy (Raso et al., 2008), United Kingdom (Okoliegbe et al., 2020), or United States (Duggan et al., 1996; Swenson and Sadikot, 2015). Nevertheless, remarkable changes in specific pairs allowed us to delineate important findings in terms of mechanisms of resistance, which was the main purpose of this work.

We first document a major role of efflux in resistance to several drugs, by evidencing quantitative correlations between the expression levels of the genes encoding efflux pumps and the increase in MICs of drug substrates, an aspect that was not examined in previous works (Bador et al., 2011, 2013; Magallon et al., 2021).

Regarding resistance to β-lactams, we did not observe any correlation between the expression of *axyB* and the MIC of ceftazidime, described as a substrate for this pump (Magallon et al., 2022), possibly because the concomitant contribution of cephalosporinase activity in resistance levels masks a potential contribution of efflux to this loss of susceptibility. This mechanism was phenotypically detected in one-third of the collection, but was not characterized at the molecular level, as it was out of the scope of this study. It is known for example that most *A. xylosoxidans* express a narrow-spectrum class D β-lactamase (Doi et al., 2008), but its role is considered marginal in resistance to cephalosporins or carbapenems (Doi et al., 2008; Amoureux et al., 2013; Papalia et al., 2020). Regarding meropenem specifically, no carbapenemase activity was detected in the collection, but we rather observed a correlation between MICs and *axyB* expression level. AxyABM has been previously shown to confer resistance to several cephalosporins and aztreonam (Bador et al., 2011) and, only very recently, to carbapenems as well (Magallon et al., 2022). In fact, previous work on carbapenems did not evidence this transport (Bador et al., 2011), probably because these authors used a strain with low meropenem MIC (0.094 mg/L), thus probably expressing the pump at a low level. This indicates the interest of also quantifying efflux pump expression level in order to better characterize their effects on susceptibility to drugs. The involvement of efflux in meropenem resistance seems to be a trait for CF isolates. We previously described that MexAB-OprM (homologous to AxyABM) plays a crucial role in meropenem resistance for *P. aeruginosa* isolates from CF (Chalhoub et al., 2016).

Aminoglycosides are considered as innately inactive against *A. xylosoxidans* (Bador et al., 2016) due to the constitutive expression of AxyXY-OprZ [expressed only in *Achromobacter* species resistant to aminoglycosides [*A. xylosoxidans*, *A. ruhlandii*, *A. dolens*, *A. insuavis*, *A. denitrificans*, *A. insolitus*, and *A. aegrifaciens* (Bador et al., 2016)]. We confirm the role of this efflux transporter in aminoglycoside resistance by demonstrating (a) the capacity of berberine to decrease aminoglycoside MIC and (b) a correlation between the expression level of *axyY* and the MICs of amikacin or tobramycin. Noteworthy, we found a few isolates that were susceptible to aminoglycosides, which could be ascribed to a particularly low level of expression of the pump. This impact of efflux on susceptibility to aminoglycosides is best seen for pairs 9, 10, 6, and 19 for which a commensurate change in MIC and in gene expression was noticed between early and late isolates. Likewise, we observed a correlation between *axyY* expression and colistin MICs, suggesting that it could be a substrate for AxyXY-OprZ. Of note, polymyxins susceptibility has been linked to MexXY-OprM/OprA expression in *P. aeruginosa* (Poole et al., 2015).

Macrolides act by inhibiting bacterial protein synthesis. A well-established resistance mechanism (in Gram-positive organisms) consists in mutations in 23S rRNA-encoding gene and in the ribosomal proteins L4 and L22 (Fyfe et al., 2016). We previously showed that both efflux and ribosomal mutations act in concert to confer high levels of resistance to macrolides in *P. aeruginosa* CF isolates (Mustafa et al., 2017). Here, we found a correlation between azithromycin MICs and the expression level of *axyB* and *axyY*, which is in line with the previously demonstrated role of efflux in macrolide resistance in *P. aeruginosa* (Morita et al., 2016). Surprisingly, however, the MexXY efflux pump attenuator berberine was able to reduce azithromycin MICs only in a limited fraction of the collection, suggesting the presence of other resistance mechanisms. Genomic analysis revealed a series of ribosomal mutations, among which A2043T, A2043G, and A2044G in *rrl*, associated with a higher level of resistance than the mutation C2596T, as previously reported in CF *P. aeruginosa* (Mustafa et al., 2017; Colque et al., 2020). Other mutations (A1284G and T1325C) have not been described so far. In addition, in two isolates with an MIC of 512 mg/L, we found mutations in the ribosomal protein 4, namely, Q65R (never described) and G69R, previously reported in macrolide-resistant *Streptococcus pneumoniae* (Clark et al., 2007; Kosowska-Shick et al., 2008), linezolid-resistant *Staphylococcus epidermidis* (Mendes et al., 2012), and CF macrolide-resistant *Burkholderia multivorans* (G70R; corresponding position) (Silva et al., 2016).

Concerning fluoroquinolones, we could not evidence a clear role of efflux in resistance, since the level of expression of *axyF* was low in most of the isolates. Of note, however, two isolates from our collection with high level of expression in *axyF* (19.2, 10.3) also show high ciprofloxacin MICs, in the absence of target mutations (10.3) or in the presence of mutations similar to those observed in a more susceptible isolate with low *axyF* expression (19.2 vs. 9.1). This is coherent with the recent description of mutations in *axyT* (putative regulator of AxyEF-OprN) associated with overexpression of *axyF* in strains harboring high ciprofloxacin MIC even in the absence of QRDR mutations (Magallon et al., 2021). In the rest of the collection, we cannot exclude that efflux-mediated resistance could be masked by the impact of target mutations on MICs. Some mutations (Q83L and D87N in the QRDR of *gyrA*) have been previously reported as hot spots in *Escherichia coli* and *P. aeruginosa* (Bagel et al., 1999; Takenouchi et al., 1999), *Stenotrophomonas maltophilia* (Zhao et al., 2015), and environmental isolates of *Achromobacter* spp. (Furlan et al., 2018), while others have never been reported in *gyrA* (L454M and T881M) and *gyrB* (I683V), but are located outside of the QRDR regions. We note here that the mutation D87N was associated with an elevated ciprofloxacin MIC (32 mg/L) in the absence of overexpression of *axyF*, while D87G was associated with even higher MICs (64–128 mg/L) in isolates overexpressing *axyF* to high levels (Magallon et al., 2021). The mutations we reported in *parC/parE* were not previously described in *A. xylosoxidans* or other species, to the best of our knowledge, but are not located in the QRDR regions. It is established that mutations in the QRDR confer higher levels of resistance to fluoroquinolones than those in other regions (Yoshida et al., 1990; Belland et al., 1994). Conversely, most of the mutations reported by Magallon et al. (2021) and considered as not relevant for resistance were not seen here. It is also interesting to note that many mutations are evidenced in the isolates from patient 18, which we suspect to belong to a new species, and may thus simply represent variation in the gene sequence.

We did not study in details resistance mechanisms to tetracyclines and chloramphenicol, but it is remarkable that all isolates with elevated MICs to chloramphenicol were overexpressing *axyF.* We cannot exclude the concomitant presence of other resistance mechanisms, but only notice that chloramphenicol is also described as a good substrate for MexEF-OprN in *P. aeruginosa* (Maseda et al., 2000).

This study suffers from some limitations. First, the number of isolates remains limited, but this is due to the still relatively low proportion of patients colonized by this bacterial genus in the collecting centers. Second, we could not establish a link between resistance development and antibiotic use in each individual patient, which could be the topic of further investigations. Third, in close relationship with the two previous limitations, we could not study in details the evolution of resistance over time because the number of samples and the period of time during which they were collected was highly variable among patients, rendering difficult a statistically meaningful analysis. Fourth, we could not confirm all our hypotheses at the molecular level because this would require the construction of a large number of deletion mutants or of complemented strains, which would represent a work by itself. In particular, we noticed that the deletion of efflux pumps in reference strains is not sufficient to cause a significant phenotypic change in susceptibility, highlighting the interest of rather working with clinical isolates that show increased levels of expression for these transporters. Specifically, the comparison of successive isogenic isolates from the same patients allowed us to unambiguously evidence the role of resistance mechanisms that were expressed in one isolate from the pair and associated with a change in MIC, partially alleviating this limitation. Fifth, the only available ATCC reference strain does not show a wild-type profile of susceptibility for all antibiotics. It appeared nevertheless to us as an adequate control, being easily available to anyone, in the absence of fully sequenced isolate from human specimen harboring a wild-type phenotype. The AXX-A strain (considered as wild type) has been recently reclassified as *A. insuavis* (NCBI:txid1003200) but has nevertheless been used as a reference sequence in our genomic analyses, in order to prevent missing the identification of some mutations associated with resistance, especially for fluoroquinolones. Lastly, the definition and evaluation of a novel cgMLST scheme for WGS-based typing of *A. xylosoxidans* should be further investigated.

Nevertheless, the present work is among the first studies to shed some light on a number of different mechanisms that most likely contribute to explain the unusually high level of resistance to conventional antibiotics in *A. xylosoxidans* or the closely related species *A. insuavis.* Our data should therefore help to better apprehend bacterial response to antibiotic exposure and adapt antibiotherapy accordingly.

## Data Availability Statement

The original contributions presented in the study are included in the article/Supplementary Material, further inquiries can be directed to the corresponding author/s.

## Author Contributions

HC, BK, and FVB: conceptualization. HC and SK: methodology, formal analysis, and investigation. SK and BK: resources. HC, SK, and FVB: writing—original draft preparation, funding acquisition. BK: writing—review and editing. BK and FVB: supervision. All authors contributed to the article and approved the submitted version.

## Conflict of Interest

The authors declare that the research was conducted in the absence of any commercial or financial relationships that could be construed as a potential conflict of interest.

## Publisher’s Note

All claims expressed in this article are solely those of the authors and do not necessarily represent those of their affiliated organizations, or those of the publisher, the editors and the reviewers. Any product that may be evaluated in this article, or claim that may be made by its manufacturer, is not guaranteed or endorsed by the publisher.

## Footnotes

1
https://mic.eucast.org/
