# Supplementary material for: Role of Efflux in Antibiotic Resistance of Achromobacter xylosoxidans and Achromobacter insuavis Isolates From Patients With Cystic Fibrosis
Source: Front Microbiol. 2022 Mar 28;13:762307. doi: 10.3389/fmicb.2022.762307 (PMC8996194; doi:10.3389/fmicb.2022.762307)
Supplement: Supplementary file 1 [file Data_Sheet_1.pdf]

Supplementary Material

Role of efflux and target mutations in antibiotic resistance of *Achromobacter xylosoxidans* and *A. insuavis* isolates from patients with cystic fibrosis

Hussein Chalhoub,[#] Stefanie Kampmeier,[#] Barbara C. Kahl and Françoise Van Bambeke

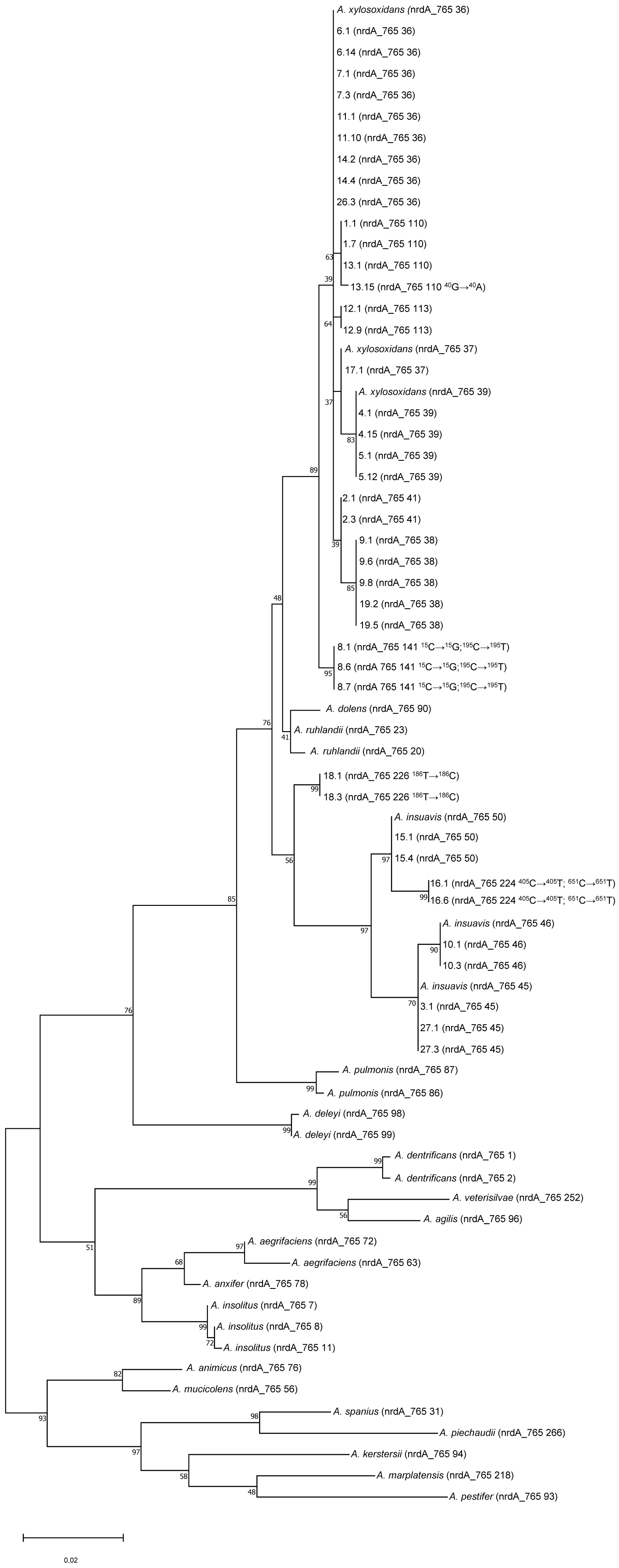

A. xylosoxidans (nrdA_765 36)
6.1 (nrdA_765 36)
6.14 (nrdA_765 36)
7.1 (nrdA_765 36)
7.3 (nrdA_765 36)
11.1 (nrdA_765 36)
11.10 (nrdA_765 36)
14.2 (nrdA_765 36)
14.4 (nrdA_765 36)
26.3 (nrdA_765 36)
1.1 (nrdA_765 110)
1.7 (nrdA_765 110)
13.1 (nrdA_765 110)
13.15 (nrdA_765 110 ^{40}G→^{40}A)
12.1 (nrdA_765 113)
12.9 (nrdA_765 113)
A. xylosoxidans (nrdA_765 37)
17.1 (nrdA_765 37)
A. xylosoxidans (nrdA_765 39)
4.1 (nrdA_765 39)
4.15 (nrdA_765 39)
5.1 (nrdA_765 39)
5.12 (nrdA_765 39)
2.1 (nrdA_765 41)
2.3 (nrdA_765 41)
9.1 (nrdA_765 38)
9.6 (nrdA_765 38)
9.8 (nrdA_765 38)
19.2 (nrdA_765 38)
19.5 (nrdA_765 38)
8.1 (nrdA_765 141 ^{15}C→^{15}G;^{195}C→^{195}T)
8.6 (nrdA 765 141 ^{15}C→^{15}G;^{195}C→^{195}T)
8.7 (nrdA 765 141 ^{15}C→^{15}G;^{195}C→^{195}T)
A. dolens (nrdA_765 90)
A. ruhlandii (nrdA_765 23)
A. ruhlandii (nrdA_765 20)
18.1 (nrdA_765 226 ^{186}T→^{186}C)
18.3 (nrdA_765 226 ^{186}T→^{186}C)
A. insuavis (nrdA_765 50)
15.1 (nrdA_765 50)
15.4 (nrdA_765 50)
16.1 (nrdA_765 224 ^{405}C→^{405}T; ^{651}C→^{651}T)
16.6 (nrdA_765 224 ^{405}C→^{405}T; ^{651}C→^{651}T)
A. insuavis (nrdA_765 46)
10.1 (nrdA_765 46)
10.3 (nrdA_765 46)
A. insuavis (nrdA_765 45)
3.1 (nrdA_765 45)
27.1 (nrdA_765 45)
27.3 (nrdA_765 45)
A. pulmonis (nrdA_765 87)
A. pulmonis (nrdA_765 86)
A. deleyi (nrdA_765 98)
A. deleyi (nrdA_765 99)
A. dentrificans (nrdA_765 1)
A. dentrificans (nrdA_765 2)
A. veterisilvae (nrdA_765 252)
A. agilis (nrdA_765 96)
A. aegrifaciens (nrdA_765 72)
A. aegrifaciens (nrdA_765 63)
A. anxifer (nrdA_765 78)
A. insolitus (nrdA_765 7)
A. insolitus (nrdA_765 8)
A. insolitus (nrdA_765 11)
A. animicus (nrdA_765 76)
A. mucicolens (nrdA_765 56)
A. spanius (nrdA_765 31)
A. piechaudii (nrdA_765 266)
A. kerstersii (nrdA_765 94)
A. marplatensis (nrdA_765 218)
A. pestifer (nrdA_765 93)
0,02

Figure S1a: Phylogenetic analysis of *Achromobacter* strains using the *nrdA*_765 typing scheme.

The evolutionary history was inferred by using the Maximum Likelihood method and General Time Reversible model (Nei and Kumar, 2000). The tree with the highest log likelihood (-3092,47) is shown. The percentage of trees in which the associated taxa clustered together is shown next to the branches. Initial tree(s) for the heuristic search were obtained automatically by applying Neighbor-Join and BioNJ algorithms to a matrix of pairwise distances estimated using the Maximum Composite Likelihood (MCL) approach, and then selecting the topology with superior log likelihood value. A discrete Gamma distribution was used to model evolutionary rate differences among sites (5 categories (+G, parameter = 0,1236)). The tree is drawn to scale, with branch lengths measured in the number of substitutions per site. This analysis involved 70 nucleotide sequences. Codon positions included were 1st+2nd+3rd+Noncoding. There were a total of 765 positions in the final dataset. Evolutionary analyses were conducted in MEGA11 (Tamura et al., 2021). Allelic match according to the nrdA_765 scheme is shown in brackets. If no exact match could be identified the nearest partial match is displayed with altered nucleobases. Closely related and all other *Achromobacter* species, for which *nrdA* typings are available are added to help better describing this collection.

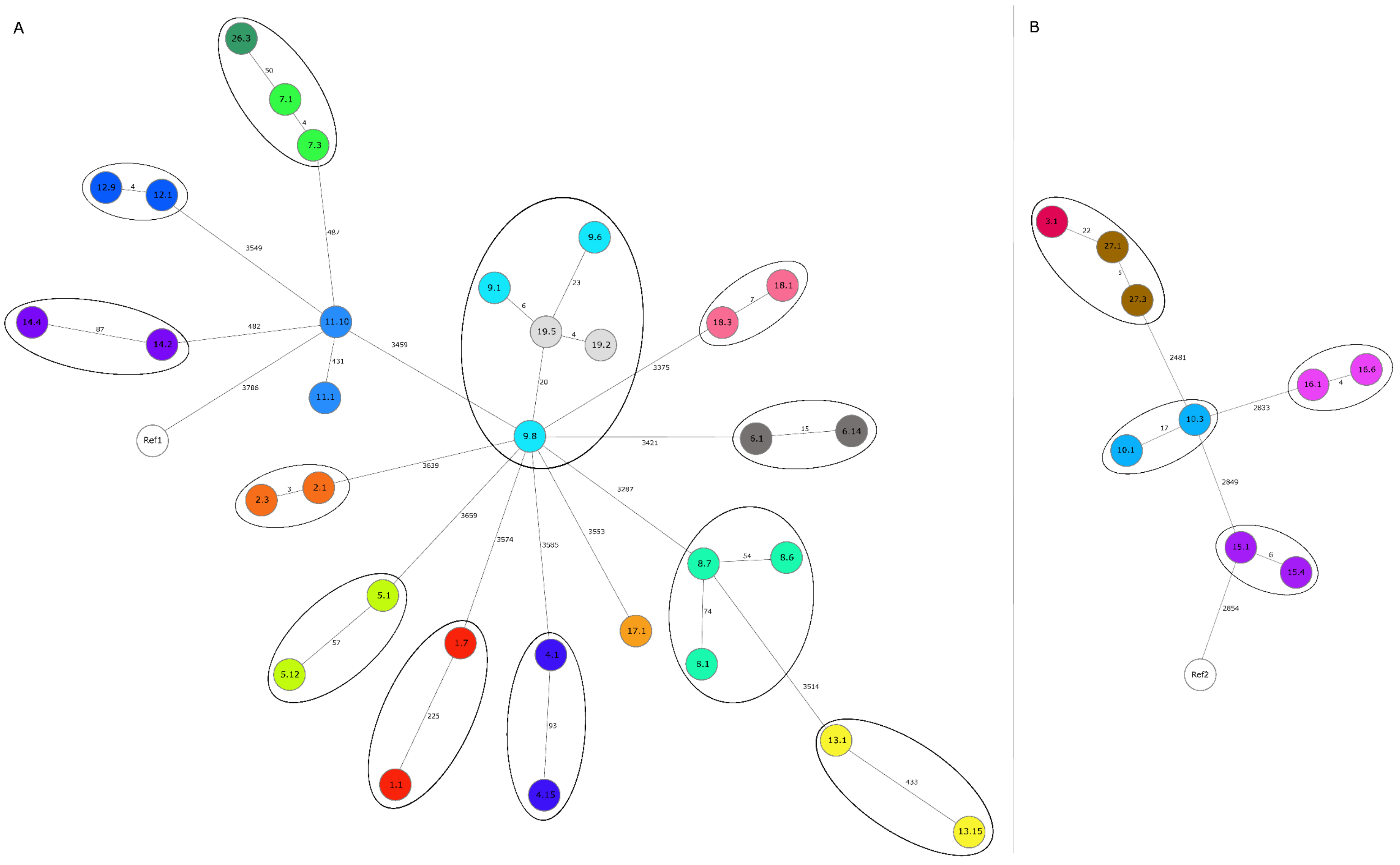
A
26.3
50
7.1
4
7.3
487
12.9
4
12.1
3549
14.4
87
14.2
482
11.10
431
11.1
3786
Ref1
3459
9.6
9.1
23
6
19.5
4
19.2
20
9.8
18.1
7
18.3
3375
6.1
15
6.14
3421
2.3
3
2.1
3639
3287
3659
3574
3585
3553
8.7
54
8.6
74
8.1
5.1
57
5.12
1.7
225
1.1
4.1
93
4.15
17.1
3514
13.1
433
13.15
B
3.1
22
27.1
5
27.3
2481
16.6
16.1
4
2833
10.3
17
10.1
2849
15.1
6
15.4
2854
Ref2

Figure S1b: Minimum spanning tree of 32 *A. xylosoxidans* (A) and 9 *A. insuavis* (B) strains isolated from 21 CF patients. Strains are displayed based on 5,778 cgMLST target genes, pairwise ignoring missing values and compared genetically to *A. xylosoxidans* (=Ref1; LN831029.1) and *A. insuavis* (=Ref2; GCA_003096315.1) reference genomes. Each circle stands for one genotype. Connecting lines indicate allelic differences between neighbour genotypes. Black borders surrounding genotypes point out genetic clusters based on allele changes over time. The same colour of circles indicates isolation of *Achromobacter* species of the same patient. Number on the circles indicate chronology.

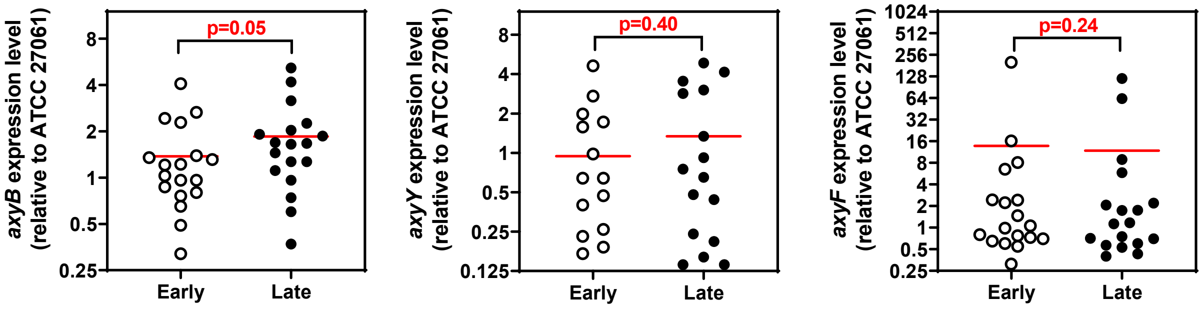

Figure S2: comparison of the expression level of *axyB*, *axyY*, or *axyF* genes in the early and late isolates from the 18 pairs collected successively in the same patients with an interval time of 1 to 11 years. Data are expressed as the expression level ($\Delta\Delta C_T$ target gene/ housekeeping gene) for each clinical isolate in comparison with the expression level measured in the reference strain ATCC 27061 (value set at 1). The red horizontal line corresponds to the mean value. Statistical analysis: Wilcoxon matched-pairs signed-rank test.

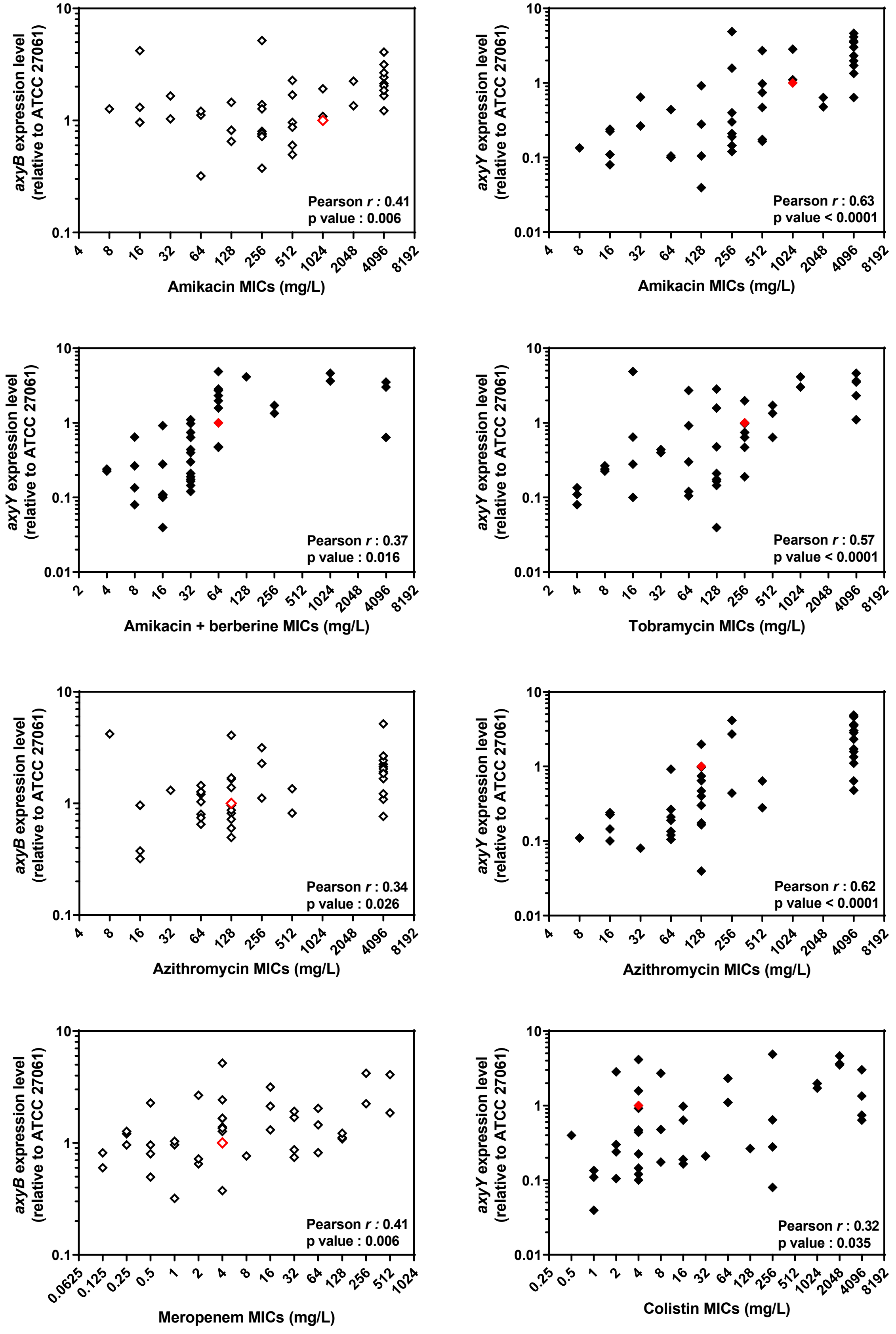
axyB expression level (relative to ATCC 27061)
Pearson r : 0.41
p value : 0.006
Amikacin MICs (mg/L)
axyY expression level (relative to ATCC 27061)
Pearson r : 0.63
p value < 0.0001
Amikacin MICs (mg/L)
axyY expression level (relative to ATCC 27061)
Pearson r : 0.37
p value : 0.016
Amikacin + berberine MICs (mg/L)
axyY expression level (relative to ATCC 27061)
Pearson r : 0.57
p value < 0.0001
Tobramycin MICs (mg/L)
axyB expression level (relative to ATCC 27061)
Pearson r : 0.34
p value : 0.026
Azithromycin MICs (mg/L)
axyY expression level (relative to ATCC 27061)
Pearson r : 0.62
p value < 0.0001
Azithromycin MICs (mg/L)
axyB expression level (relative to ATCC 27061)
Pearson r : 0.41
p value : 0.006
Meropenem MICs (mg/L)
axyY expression level (relative to ATCC 27061)
Pearson r : 0.32
p value : 0.035
Colistin MICs (mg/L)

Figure S3: Correlation between the expression level of *axyB, axyY* or *axyF* and the MIC of antibiotics. These graphs show only those data for which significant correlations were evidenced. See Supplementary Table S2 for the whole set of data. Correlations with *axyB* levels are shown with open symbols; those with *axyY,* with closed symbols; those with *axyF*, with grey symbols. Data are shown as the expression level (ΔΔCT target gene/housekeeping gene) for each clinical isolate in comparison with the expression level measured in the reference strain ATCC 27061 (value set at 1; illustrated in the graphs by the red symbols).

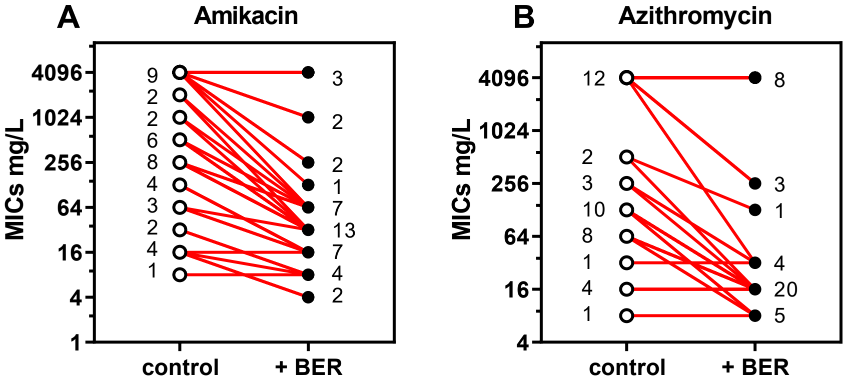

Figure S4: Influence of active efflux on amikacin, tobramycin and azithromycin activity. MICs of amikacin (A) and azithromycin (B) in the absence (control) of in the presence of 128 mg/L berberine (BER) for the 41 clinical isolates. The red lines joint data points for the same isolates. Figures on the left and on the right show the number of isolates presenting each MIC value.

Table S1: Primers and conditions* used for real-time PCR

**Efflux systems**	
axyB-Fwd	5'- AGGTGATCGAGCAGCAGATG -3'
axyB -Rev	5'- AACGTCAGCGTGATGGACATG -3'
axyY-Fwd	5'- TGGTGTTCTGCGTGATGTAC-3'
axyY-Rev	5'- ACATCGTCAGCACGTTGATC -3'
axyF-Fwd	5'- TTCGCTGCTGGACAACAAG-3'
axyF-Rev	5'- TCGTATTCGATGCGGTATTCC-3'
**Housekeeping genes**	
16S-Fwd	5'- ACAAGCGGTGGATGATGTG -3'
16S-Rev	5'- ATCTCTTCGGCATTCCAGACATG-3'

*Two min of cDNA denaturation at 98 °C, followed by 2 steps (39 cycles) of 10 s at 98 °C, 60 s at 64.4 °C. A melt curve was run at the end of the real-time PCR cycles, to check for the presence of a unique PCR reaction product.

Table S2: Relationship between efflux genes expression (mean of n=2) and antibiotic MICs in 41 *Achromobacter* CF isolates (versus the reference strain ATCC 27061)

Parameter **for AMK MICs versus gene expression levels**	***axyB* expression levels**	***axyY* expression levels**	***axyF* expression levels**
**Number of XY Pairs**	42	42	42
**Pearson r**	0.4155	0.6289	-0.2044
**95% confidence interval**	0.1276 to 0.6388	0.4018 to 0.7832	-0.4787 to 0.1062
**P value (two-tailed)**	0.0062	P<0.0001	0.1940
**P value summary**	**	***	ns
**Is the correlation significant? (alpha=0.05)**	Yes	Yes	No
**R squared**	0.1726	0.3956	0.04180

Parameter **for AMK+BER MICs versus gene expression levels**	***axyB* expression levels**	***axyY* expression levels**	***axyF* expression level**
**Number of XY Pairs**	42	42	42
**Pearson r**	0.09429	0.3720	-0.1039
**95% confidence interval**	-0.2159 to 0.3872	0.07670 to 0.6073	-0.3954 to 0.2066
**P value (two-tailed)**	0.5526	0.0153	0.5124
**P value summary**	ns	*	ns
**Is the correlation significant? (alpha=0.05)**	No	Yes	No
**R squared**	0.008890	0.1384	0.01080

Parameter for **AZI MICs versus gene expression levels**	***axyB* expression levels**	***axyY* expression levels**	***axyF* expression level**
**Number of XY Pairs**	42	42	42
**Pearson r**	0.3422	0.6241	-0.1839
**95% confidence interval**	0.04260 to 0.5853	0.3950 to 0.7801	-0.4620 to 0.1273
**P value (two-tailed)**	0.0266	P<0.0001	0.2438
**P value summary**	*	***	ns
**Is the correlation significant? (alpha=0.05)**	Yes	Yes	No
**R squared**	0.1171	0.3894	0.03380

Parameter for **AZI+BER MICs versus gene expression levels**	***axyB* expression levels**	***axyY* expression levels**	***axyF* expression level**
**Number of XY Pairs**	42	42	42
**Pearson r**	0.05779	0.1873	-0.1428
**95% confidence interval**	-0.2506 to 0.3555	-0.1238 to 0.4648	-0.4282 to 0.1685
**P value (two-tailed)**	0.7162	0.2350	0.3670
**P value summary**	ns	ns	ns
**Is the correlation significant? (alpha=0.05)**	No	No	No
**R squared**	0.003340	0.03507	0.02039

Parameter for **TMO MICs versus gene expression levels**	***axyB* expression levels**	***axyY* expression levels**	***axyF* expression level**
**Number of XY Pairs**	42	42	42
**Pearson r**	0.1170	0.2069	-0.2559
**95% confidence interval**	-0.1939 to 0.4065	-0.1036 to 0.4807	-0.5195 to 0.05220
**P value (two-tailed)**	0.4607	0.1886	0.1019
**P value summary**	ns	ns	ns
**Is the correlation significant? (alpha=0.05)**	No	No	No

Parameter **for PIP-TZB (4 mg/L) MICs versus gene expression levels**	***axyB* expression levels**	***axyY* expression levels**	***axyF* expression level**
**Number of XY Pairs**	42	42	42
**Pearson r**	0.1345	0.03159	-0.1301
**95% confidence interval**	-0.1767 to 0.4213	-0.2751 to 0.3324	-0.4176 to 0.1811
**P value (two-tailed)**	0.3958	0.8426	0.4116
**P value summary**	ns	ns	ns
**Is the correlation significant? (alpha=0.05)**	No	No	No
**R squared**	0.01809	0.0009978	0.01692

Parameter **for MEM MICs versus gene expression levels**	***axyB* expression levels**	***axyY* expression levels**	***axyF* expression level**
**Number of XY Pairs**	42	42	42
**Pearson r**	0.4150	0.1463	-0.08230
**95% confidence interval**	0.1270 to 0.6384	-0.1651 to 0.4311	-0.3769 to 0.2274
**P value (two-tailed)**	0.0063	0.3553	0.6043
**P value summary**	**	ns	ns
**Is the correlation significant? (alpha=0.05)**	Yes	No	No
**R squared**	0.1722	0.02139	0.006774

Parameter **for CIP MICs versus gene expression levels**	***axyB* expression levels**	***axyY* expression levels**	***axyF* expression level**
**Number of XY Pairs**	42	42	42
**Pearson r**	0.09325	0.09895	0.2489
**95% confidence interval**	-0.2169 to 0.3863	-0.2114 to 0.3912	-0.05957 to 0.5140
**P value (two-tailed)**	0.5569	0.5330	0.1119
**P value summary**	ns	ns	ns
**Is the correlation significant? (alpha=0.05)**	No	No	No
**R squared**	0.008696	0.009791	0.06197

Parameter **for TIC MICs versus gene expression levels**	***axyB* expression levels**	***axyY* expression levels**	***axyF* expression level**
**Number of XY Pairs**	42	42	42
**Pearson r**	0.2834	0.03582	-0.1719
**95% confidence interval**	-0.02250 to 0.5408	-0.2711 to 0.3362	-0.4523 to 0.1394
**P value (two-tailed)**	0.0689	0.8218	0.2764
**P value summary**	ns	ns	ns
**Is the correlation significant? (alpha=0.05)**	No	No	No
**R squared**	0.08034	0.001283	0.02955

Parameter **for TIC-AVI (32 mg/L) MICs versus gene expression levels**	***axyB* expression levels**	***axyY* expression levels**	***axyF* expression level**
**Number of XY Pairs**	42	42	42
**Pearson r**	0.03222	0.09607	-0.1608
**95% confidence interval**	-0.2745 to 0.3330	-0.2142 to 0.3887	-0.4432 to 0.1505
**P value (two-tailed)**	0.8395	0.5450	0.3089
**P value summary**	ns	ns	ns
**Is the correlation significant? (alpha=0.05)**	No	No	No
**R squared**	0.001038	0.009229	0.02587

Parameter **for CAZ MICs versus gene expression levels**	***axyB* expression levels**	***axyY* expression levels**	***axyF* expression level**
**Number of XY Pairs**	42	42	42
**Pearson r**	-0.04817	0.1270	-0.1215
**95% confidence interval**	-0.3471 to 0.2596	-0.1841 to 0.4150	-0.4103 to 0.1895
**P value (two-tailed)**	0.7619	0.4230	0.4435
**P value summary**	ns	ns	ns
**Is the correlation significant? (alpha=0.05)**	No	No	No
**R squared**	0.002321	0.01612	0.01475

Parameter **for TOB MICs versus gene expression levels**	***axyB* expression levels**	***axyY* expression levels**	***axyF* expression level**
**Number of XY Pairs**	42	42	42
**Pearson r**	0.1731	0.5752	-0.1341
**95% confidence interval**	-0.1381 to 0.4533	0.3286 to 0.7483	-0.4209 to 0.1772
**P value (two-tailed)**	0.2728	P<0.0001	0.3973
**P value summary**	ns	***	ns
**Is the correlation significant? (alpha=0.05)**	No	Yes	No
**R squared**	0.02998	0.3308	0.01797

Parameter **for CST MICs versus gene expression levels**	***axyB* expression levels**	***axyY* expression levels**	***axyF* expression level**
**Number of XY Pairs**	42	42	42
**Pearson r**	0.1765	0.3338	-0.1353
**95% confidence interval**	-0.1347 to 0.4561	0.03312 to 0.5790	-0.4219 to 0.1760
**P value (two-tailed)**	0.2634	0.0308	0.3930
**P value summary**	ns	*	ns
**Is the correlation significant? (alpha=0.05)**	No	Yes	No
**R squared**	0.03116	0.1114	0.01830

Parameter **for CHL MICs versus gene expression levels**	***axyB* expression levels**	***axyY* expression levels**	***axyF* expression level**
**Number of XY Pairs**	42	42	42
**Pearson r**	-0.1932	-0.2655	0.6610
**95% confidence interval**	-0.4695 to 0.1177	-0.5269 to 0.04179	0.4468 to 0.8035
**P value (two-tailed)**	0.2203	0.0892	<0.0001
**P value summary**	ns	ns	***
**Is the correlation significant? (alpha=0.05)**	No	No	Yes
**R squared**	0.03731	0.07050	0.4369

Parameter **for DOX MICs versus gene expression levels**	***axyB* expression levels**	***axyY* expression levels**	***axyF* expression level**
**Number of XY Pairs**	42	42	42

**Pearson r**	0.04138	-0.01640	-0.1684
**95% confidence interval**	-0.2697 to 0.3446	-0.3224 to 0.2927	-0.4526 to 0.1469
**P value (two-tailed)**	0.7973	0.9189	0.2926
**P value summary**	ns	ns	ns
**Is the correlation significant? (alpha=0.05)**	No	No	No
**R squared**	0.001712	0.0002690	0.02836

Table S3: Resistance mechanisms to azithromycin, including ribosomal mutations in *rpl4, rpl22, rrl* genes and expression of efflux pumps. *A. insuavis* AXX-A (MIC, 64 mg/L) and *A. xylosoxidans* ATCC27061 (MIC, 128 mg/L) are used as references.

Strain identification[a]		AZI MIC (mg/L)		Ribosomal mutations[b]			Gene expression level	
Strain	Isolation year	-BER[c]	+BER	*rpl4*	*rpl22*	*rrl*	*axyB*[g]	*axyY*[g]
ATCC 27061		128	16	Silent mutations (T96C, T123C, T411C)	T134C (V45A) Silent mutations (T78C, T189C)	C144T, T275C, A371G, G1012A, C1172T, T1178_A1179del, G1180A, A1385G, T1402C, A1471G, A2792G	1	1
16.1[h]	2016	16	16	Silent mutation (C219G)	-	A1385G, T1402C, A1471G, T1519C	0.96	0.23
16.6 [h]	2017	16	16	Silent mutation (C219G)	-	Missing data	0.96	0.24
15.1[h]	2009	16	16	-	-	Missing data	0.32	0.10
15.4[h]	2013	16	16	-	-	Missing data	0.37	0.14
6.1	2006	128	8	Silent mutations (T96C, T123C, C444T)	T134C (V45A) Silent mutations (T78C, T189C)	C141G, G148C, T275C, A371G, G1012A, T1178_A1179del, A1385G, T1402C, A1471G, T1519C, A2792G	4.08	1.98
6.14	2017	8	8	Silent mutations (T96C, T123C, C444T)	T134C (V45A) Silent mutations (T78C, T189C)	C141G, G148C, T275C, A371G, G1012A, T1178_A1179del, A1385G, T1402C, A1471G, T1519C, A2792G	4.19	0.11
13.1	2009	128	16	Silent mutations (T96C, T123C, T411C)	T134C (V45A) Silent mutations (T78C, T189C	T275C, G371A, G1012A, T1178_A1179del, A1385G, T1402C, A1471G, T1519C, A2792G	0.87	0.98
27.1[h]	2010	64	16	Silent mutations (T411C)	T134C (V45A)	A120G, G1012A, T1178_A1179del, A1385G, T1402C	1.21	0.11
27.3[h]	2013	64	16	Silent mutations (T411C)	T134C (V45A)	A120G, G1012A, T1178_A1179del, A1385G, T1402C	1.27	0.12
10.1[h]	2010	32	32	Silent mutations (T411C)	T134C (V45A)	A120G, G1012A, T1178_A1179del, A1385G, T1402C, A1471G	1.31	0.08
10.3[h]	2011	256	32	Silent mutations (T411C)	T134C (V45A)	A120G, G1012A, T1178_A1179del, A1385G, T1402C, A1471G	1.11	0.44
1.1	2011	256	32	Silent mutations (T96C,	T134C (V45A) Silent mutations	C144T, T275C, A371G, G1012A, T1178_A1179del, A1385G, T1402C,	2.28	2.72

				T123C, T411C)	(T78C, T189C)	A1471G, T1519C, A2792G		
1.7	2015	256	16	Silent mutations (T96C, T123C, T411C)	T134C (V45A) Silent mutations (T78C, T189C)	T275C, A371G, G1012A, T1178_A1179del, A1385G, T1402C, A1471G, T1519C, A2792G	3.16	4.15
7.1	2014	128	16	Silent mutations (T96C, T123C, T411C)	T134C (V45A) Silent mutations (T78C, T189C)	C144T, T275C, A371G, G1012A, T1175A, T1178_A1179del, A1385G, T1402C, A2792G	0.96	0.47
26.3	2017	128	16	Silent mutations (T96C, T123C, T411C)	T134C (V45A) Silent mutations (T78C, T189C)	C144T, T275C, A371G, G1012A, T1175A, T1178_A1179del, A1385G, T1402C, A2792G	0.72	0.30
17.1	2014	>2048	>2048	Silent mutations (T96C, T123C, T411C, C414T)	T134C (V45A) Silent mutations (T78C, T189C)	T275C, A371G, G1012A, T1175A, T1178_A1179del, A1385G, T1402C, A1471G, T1519C, A2792G	1.09	1.11
4.1	2010	>2048	>2048	Silent mutations (T96C, T123C, T411C, C414T)	T134C (V45A) Silent mutations (T78C, T189C)	T275C, A371G, G1012A, C1170T, T1178_A1179del, G1180A, A1385G, T1402C, A1471G, T1519C, **A2043T**[f], A2792G,	2.43	1.72
4.15	2017	>2048	>2048	Silent mutations (T96C, T123C, T411C, C414T)	T134C (V45A) Silent mutations (T78C, T189C)	C144T, T275C, A371G, G1012A, C1170T, T1178_A1179del, A1385G, T1402C, A1471G, T1519C, **A2043T**, A2792G	1.67	1.34
5.1	2007	512	128	Silent mutations (T96C, T123C, T411C, C414T) **A194G (Q65R) uncharged to charged**	T134C (V45A) Silent mutations (T78C, T189C)	C144T, T275C, A371G, G1012A, C1172T, T1178_A1179del, G1180A, A1385G, T1402C, A1471G, T1519C, A2792G	1.35	0.64
5.12	2015	>2048	>2048	Silent mutations (T96C, T123C, T411C, C414T)	T134C (V45A) Silent mutations (T78C, T189C)	C144T, T275C, A371G, G1012A, C1172T, T1178_A1179del, G1180A, A1385G, T1402C, A1471G, T1519C, **A2043G**[f], A2792G	2.25	0.48

9.1	2010	64	8	Silent mutations (T96C, T123C, T411C)	T134C (V45A) Silent mutations (T78C, T189C)	C144T, T275C, A371G, G1012A, C1172T, T1178_A1179del, G1180A, A1385G, T1402C, A1471G, T1519C, A2792G	1.03	0.26
9.6	2012	512	16	Silent mutations (T96C, T123C, T411C), **G205C (G69R[d]) uncharged to charged**	T134C (V45A) Silent mutations (T78C, T189C)	C144T, T275C, A371G, G1012A, C1172T, T1178_A1179del, G1180A, A1385G, T1402C, A1471G, T1519C, A2792G	0.82	0.28
9.8	2013	>2048	32	Silent mutations (T96C, T123C, T411C)	T134C (V45A) Silent mutations (T78C, T189C)	C144T, T275C, A371G, G1012A, T1178_A1179del, G1180A, A1385G, T1402C, A1471G, T1519C, **A2044G[f],** A2792G	5.17	4.87
8.1	2010	>2048	256	Silent mutation (T96C, T123C, C330T, T411C)	T134C (V45A) Silent mutations (T78C, T189C)	C144T, T275C, A371G, G1012A, C1172T, T1178_A1179del, G1180A, A1385G, T1402C, A1471G, T1519C, **C2596T[e],** A2792G.	2.66	4.63
8.6	2014	>2048	256	Silent mutation (T96C, T123C, C330T, T411C)	T134C (V45A) Silent mutations (T78C, T189C)	C144T, T275C, A371G, G1012A, C1172T, T1178_A1179del, G1180A, A1385G, T1402C, A1471G, T1519C, **C2596T[e],** A2792G.	2.13	3.64
8.7	2015	>2048	256	Silent mutation (T96C, T123C, C330T, T411C)	T134C (V45A) Silent mutations (T78C, T189C)	C144T, T275C, A371G, G1012A, C1172T, T1178_A1179del, G1180A, A1385G, T1402C, A1471G, T1519C, **C2596T[e],** A2792G.	2.03	3.53
11.1	2010	>2048	>2048	Silent mutations (T96C, T123C, T411C)	T134C (V45A) Silent mutations (T78C, T189C)	C144T, T275C, A371G, G1012A, T1175A, T1178_A1179del, **T1325C**, A1385G, T1402C, **A2043G,** A2792G	1.22	0.64
11.10	2015	>2048	>2048	Silent mutations (T96C, T123C, T411C)	T134C (V45A) Silent mutations (T78C, T189C)	C144T, T275C, A371G, G1012A, T1175A, T1178_A1179del, **A1284G**, A1385G, T1402C, **A2044G,** A2792G	1.86	3.02

14.2	2014	>2048	>2048	Silent mutations (T96C, T123C, T411C)	T134C (V45A) Silent mutations (T78C, T189C)	C144T, T275C, A371G, G1012A, T1175A, T1178_A1179del, A1385G, T1402C, **A2043G,** A2792G	0.76	1.58
14.4	2016	>2048	>2048	Silent mutations (T96C, T123C, T411C)	T134C (V45A) Silent mutations (T78C, T189C)	C144T, T275C, A371G, G1012A, T1175A, T1178_A1179del, A1385G, T1402C, **A2043G,** A2792G	1.91	2.84

[a]strain numbering: first figure, patient identification number; second figure, isolate number in this patient (late isolate over the whole period of sampling; see also Table S3). The strains gathered in the same quadrant without inside borders are successive isolates from the same patient. They are classified in order to show close from one another those which share the same mutations, ordered by increasing MICs.

[b] Reference sequence is that of *A. insuavis* AXX-A. (-) : same sequence as AXX-A. Mutations highlighted in blue are found in *A. xylosoxidans* ATCC 27061 when comparing its sequence with that *of A. insuavis* AXX-A which has a AZI MIC of 64 mg/L. Mutations in green have not been previously reported; mutations in red are found only in isolates with MIC ≥ 512 mg/L.

[c] used at 128 mg/L

[d] previously reported in macrolide-resistant *Streptococcus pneumoniae* (Clark et al., 2007; Kosowska-Shick et al., 2008) in linezolid-resistant *Staphylococcus epidermidis* (Mendes et al., 2012) and in macrolide-resistant *Burkholderia multivorans* form patient with CF (corresponding to position 70, G70R)(Silva et al., 2016).

[e] the sequence of AXX-A is numbered based on its alignment with that of the corresponding sequence in *P. aeruginosa* to facilitate the identification of previously described mutations.

[f] A2043T, A2043G, A2044G induced higher levels of resistance to azithromycin than C2596T in *P. aeruginosa* (Mustafa et al., 2017).

[g] as determined by qPCR.

[h]*A. insuavis*

Table S4: Resistance mechanisms to ciprofloxacin, including expression levels of *axyF* and mutations in *gyrA*, *gyrB*, *parC*, and *parE*. *A. insuavis* AXX-A (MIC, 1 mg/L) and *A. xylosoxidans* ATCC27061 (MIC, 4-8 mg/L) are used as references.

Sample identification[a]		CIP MIC (mg/L)[b]	*axyF* expression levels[c]	Mutations[d]			
Strains	Isolation year			*gyrA*	*gyrB*	*parC*	*parE*
ATCC 27061	ref	4-8	1	T204S, I222V, V488I, A491T, D531E, 866_868insGQE, D869E	N188T, I190V, A258T, A387G, S581A, E629D, S630T, R639K, V649I, E662A, V689I, R699K, R711Q, A714V, E720D, N726T, E759D	H7Q, G281C, V382I, V384A, K421N, V437A, E447D, A474E, S482A, F508Y, V536I, S591A, L693V, V741A	V11I, Y27H, Q32L, F132Y, E219Q, S335A, S463T, N507H, A555S, S557A
3.1	2012	1	0.7	G879A	Y64W, A258S, R639K	G281C, N392H, F508Y	-
15.1 [e]	2009	4	0.6	-	N592S, A631T, A633S, A634T, R639K	-	-
16.1	2016	16	201	**L454M**	-	-	-
18.1-18.3	2009-2011	4-8	1.1	T204S, I222V, T527S, D531E	N11T, G12S, N188T, I190V, A258T, A387G, N592G, S609A, E629D, S630T, R639K, E662A, V689I, R699K, R711Q, E720D, N726T, E759D	H7Q, V382I, V384A, N392H, K421N, V437A, E441Q, E447D, A474E, S482T, V536I, S591A, L693V, V741A, K764R	V11I, Y27H, Q32L, V41I, F132Y, A150T, S335A, S463T, N507H, A555S, S557A, T593S
9.1	2010	4	6.5	T204S, I222V, V488I, A491T, D531E, 866_868insGQE, D869E[f]	N188T, I190V, A258T, A387G, S581A, E629D, S630T, R639K, V649I, E662A, V689I, R699K, R711Q, A714V, E720D, N726T, E759D	H7Q, G281C, V382I, V384T, V417L, K421N, V437A, E447D, A474E, S482A, F508Y, V536I, L693V, V741A	V11I, Y27H, Q32L, F132Y, E219Q, S335A, S463T, N507H, A555S, S557A
19.2	2009	32	16.2	T204S, I222V, V488I, A491T, D531E, 866_868insGQE, D869E[f]	N188T, I190V, A258T, A387G, S581A, E629D, S630T, R639K, V649I, E662A, V689I, R699K, R711Q, A714V, E720D, N726T, E759D	H7Q, G281C, V382I, V384T, V417L, K421N, V437A, E447D, A474E, S482A, F508Y, V536I, L693V, V741A	V11I, Y27H, Q32L, F132Y, E219Q, S335A, S463T, N507H, A555S, S557A
17.1	2014	16	0.9	T204S, I222V, V488I, A491T, D531E, 866_868insGQE, D869E[f], **T881M**	N188T, I190V, A258T, A387G, S581A, E629D, S630T, R639K, V649I, E662A, V689I, R699K, R711Q, A714V, E720D, N726T, E759D	H7Q, G281C, V382I, V384A, K421N, V437A, E447D, A474E, S482A, F508Y, V536I, S591A, L693V, V741A -	V11I, Y27H, Q32L, F132Y, E219Q, S335A, S463T, N507H, A555S, S557A

4.1	2010	4	2.2	T204S, I222V, D531E, 866_868insGQE, D869E[f]	N188T, I190V, A258T, A387G, S581A, E629D, S630T, R639K, V649I, E662A, V689I, R699K, R711Q, A714V, E720D, N726T, E759D	H7Q, G281C, V382I, V384T, K421N, V437A, E447D, A474E, S482A, F508Y, V536I, S591A, L693V, V741A	V11I, Y27H, Q32L, F132Y, E219Q, S335A, S463T, N507H, A555S, S557A
4.15	2017	16	2.2	**Q83L**<br><br>T204S, I222V, D531E, ins 866_868, D869E[f]	N188T, I190V, A258T, A387G, S581A, E629D, S630T, R639K, V649I, E662A, V689I, R699K, R711Q, A714V, E720D, N726T, E759D	H7Q, G281C, V382I, V384T, K421N, V437A, E447D, A474E, S482A, F508Y, V536I, S591A, L693V, V741A	V11I, Y27H, Q32L, F132Y, E219Q, S335A, S463T, N507H, A555S, S557A
8.1	2010	8	0.7	T204S, I222V, A491T, D531E, M706V, ins 866_868, D869E[f]	N188T, I190V, A258T, A387G, S581A, E629D, S630T, R639K, V649I, E662A, V689I, R699K, R711Q, A714V, E720D, N726T, E759D	H7Q, G281C, V382I, V384T, N392H K421N, V437A, E447D, A474E, S482A, F508Y, V536I, S591A, L693V, V741A	V11I, Y27H, Q32L, F132Y, E219Q, S335A, N507H, A555S, S557A
8.6	2014	32	0.7	**D87N**, T204S, I222V, A491T, D531E, M706V, ins 866_868, D869E[f]	N188T, I190V, A258T, A387G, S581A, E629D, S630T, R639K, V649I, E662A, V689I, R699K, R711Q, A714V, E720D, N726T, E759D	H7Q, G281C, V382I, V384T, N392H K421N, V437A, E447D, A474E, S482A, F508Y, V536I, S591A, L693V, V741A	V11I, Y27H, Q32L, F132Y, E219Q, S335A, N507H, A555S, S557A
2.1	2016	4	0.8	T204S, I222V, V488I, A491T, D531E, 866_868insGQE, D869E[f]	N188T, I190V, A258T, A387G, S581A, E629D, S630T, R639K, V649I, E662A, V689I, R699K, R711Q, A714V, E720D, N726T, E759D	H7Q, G281C, V382I, V384T, K421N, V437A, E447D, A474E, S482A, F508Y, V536I, S591A, L693V, V741A	V11I, Y27H, Q32L, F132Y, E219Q, S335A, S463T, N507H, A555S, S557A
5.1	2007	16	0.7	T204S, I222V, V488I, A491T, D531E, 866_868insGQE, D869E[f]	N188T, I190V, A258T, A387G, S581A, E629D, S630T, R639K, V649I, E662A, **I683V**, V689I, R699K, R711Q, A714V, E720D, N726T, E759D	H7Q, G281C, V382I, V384T, K421N, V437A, E447D, A474E, S482A, F508Y, V536I, S591A, L693V, V741A	V11I, Y27H, Q32L, F132Y, E219Q, S335A, N507H, A555S, S557A
5.12	2015	32	0.7	T204S, I222V, V488I, A491T, D531E, 866_868insGQE, D869E[f]	N188T, I190V, A258T, A387G, S581A, E629D, S630T, R639K, V649I, E662A, **I683V,** V689I,	H7Q, G281C, V382I, V384T, K421N, V437A, E447D, A474E, S482A, F508Y,	V11I, Y27H, Q32L, F132Y, E219Q, S335A, N507H, A555S, S557A

					R699K, R711Q, A714V, E720D, N726T, E759D	V536I, S591A, L693V, V741A	
10.1	2010	4	0.7	G879A[f]	Y64W, A258S, R639K	G281C, N392H, F508Y	-
10.3[e]	2011	128	63	G879A[f]	Y64W, A258S, R639K	G281C, N392H, F508Y	-

[a]strain numbering: first figure, patient identification number; second figure, isolate number in this patient (late isolate over the whole period of sampling; see also Table S3). The strains are classified in order to show close from one another those which share the same mutations, ordered by increasing MICs. Strains gathered in the same quadrant without inside borders can be compared as showing additional mutations associated with higher MICs in the presence or absence of *axyF* overexpression.

[b] CLSI breakpoints (mg/L) for CIP: S ≤ 1; [c] as determined by qPCR.

[d] Reference sequence is that of *A. insuavis* AXX-A. **(-)**: same sequence as AXX-A. Mutations found in isolates with CIP MIC ≤ 1mg/L, ≥ 4, or 16 mg/L are highlighted in blue, green, or red colour respectively. Several mutations highlighted in green are also found in *A. xylosoxidans* ATCC 27061 when comparing its sequence with that *of A. insuavis* AXX-A which has a CIP MIC of 1 mg/L and could be associated with resistance.

[e]*A. insuavis*

[f] numbering based on the ATCC 27061 sequence (because located after the insertion of 3 aminoacids in this strain as compared to AXX-A)

References

Clark, C. L., Kosowska-Shick, K., Ednie, L. M., and Appelbaum, P. C. (2007). Capability of 11 antipneumococcal antibiotics to select for resistance by multistep and single-step methodologies. *Antimicrob. Agents Chemother.* 51, 4196-4201.

Kosowska-Shick, K., Clark, C., Credito, K., Dewasse, B., Beachel, L., Ednie, L., and Appelbaum, P. C. (2008). In vitro capability of faropenem to select for resistant mutants of Streptococcus pneumoniae and Haemophilus influenzae. *Antimicrob. Agents Chemother.* 52, 748-752.

Mendes, R. E., Deshpande, L. M., Costello, A. J., and Farrell, D. J. (2012). Molecular epidemiology of Staphylococcus epidermidis clinical isolates from U.S. hospitals. *Antimicrob. Agents Chemother.* 56, 4656-4661.

Mustafa, M. H., Khandekar, S., Tunney, M. M., Elborn, J. S., Kahl, B. C., Denis, O., Plesiat, P., Traore, H., Tulkens, P. M., Vanderbist, F., and Van Bambeke, F. (2017). Acquired resistance to macrolides in Pseudomonas aeruginosa from cystic fibrosis patients. *Eur. Respir. J.* 49, 1601847.

Nei, M. and Kumar, S. (2000). Molecular Evolution and Phylogenetics. 1-348.

Silva, I. N., Santos, P. M., Santos, M. R., Zlosnik, J. E. A., Speert, D. P., Buskirk, S. W., Bruger, E. L., Waters, C. M., Cooper, V. S., and Moreira, L. M. (2016). Long-Term Evolution of Burkholderia multivorans during a Chronic Cystic Fibrosis Infection Reveals Shifting Forces of Selection. *mSystems.* 1, e00029-16.

Tamura, K., Stecher, G., and Kumar, S. (2021). MEGA11: Molecular Evolutionary Genetics Analysis Version 11. *Mol. Biol. Evol.* 38, 3022-3027.
